# Diverse pathways in GPCR-mediated activation of Ca^2+^ mobilization in HEK293 cells

**DOI:** 10.1016/j.jbc.2024.107882

**Published:** 2024-10-10

**Authors:** Francesco De Pascali, Asuka Inoue, Jeffrey L. Benovic

**Affiliations:** 1 Department of Biochemistry and Molecular Biology, Sidney Kimmel Medical College, Thomas Jefferson University, Philadelphia, Pennsylvania, USA; 2 Graduate School of Pharmaceutical Sciences, Tohoku University, Sendai, Miyagi, Japan; 3 Graduate School of Pharmaceutical Sciences, Kyoto University, Kyoto, Japan

**Keywords:** β_2_-adrenergic receptor, Ca^2+^ mobilization, G protein-coupled receptor, heterotrimeric G proteins, prostaglandin E receptor

## Abstract

G protein-coupled receptors transduce extracellular stimuli into intracellular signaling. Ca^2+^ is a well-known second messenger that can be induced by G protein-coupled receptor activation through the primary canonical pathways involving Gα_q_- and Gβγ-mediated activation of phospholipase C-β (PLCβ). While some G_s_-coupled receptors are shown to trigger Ca^2+^ mobilization, underlying mechanisms remain elusive. Here, we evaluated whether G_s_-coupled receptors including the β_2_-adrenergic receptor (β_2_AR) and the prostaglandin EP_2_ and EP_4_ receptors (EP_2_R and EP_4_R) that are endogenously expressed in human embryonic kidney 293 (HEK293) cells utilize common pathways for mediating Ca^2+^ mobilization. For the β_2_AR, we found an essential role for G_q_ in agonist-promoted Ca^2+^ mobilization while genetic or pharmacological inhibition of G_s_ or G_i_ had minimal effect. β-agonist-promoted Ca^2+^ mobilization was effectively blocked by the G_q_-selective inhibitor YM-254890 and was not observed in ΔGα_q/11_ or ΔPLCβ cells. Bioluminescence resonance energy transfer analysis also suggests agonist-dependent association of the β_2_AR with G_q_. For the EP_2_R, which couples to G_s_, agonist treatment induced Ca^2+^ mobilization in a pertussis toxin-sensitive but YM-254890-insensitive manner. In contrast, EP_4_R, which couples to G_s_ and G_i_, exhibited Ca^2+^ mobilization that was sensitive to both pertussis toxin and YM-254890. Interestingly, both EP_2_R and EP_4_R were largely unable to induce Ca^2+^ mobilization in ΔGα_s_ or ΔPLCβ cells, supporting a strong dependency on G_s_ signaling in HEK293 cells. Taken together, we identify differences in the signaling pathways that are used to mediate Ca^2+^ mobilization in HEK293 cells where the β_2_AR primarily uses G_q_, EP_2_R uses G_s_ and G_i_, and EP_4_R uses G_s_, G_i_, and G_q_.

G protein-coupled receptors (GPCRs) are dynamic proteins that transduce extracellular stimuli into intracellular signaling to ultimately induce a cellular response. GPCR activation by an agonist promotes interaction with heterotrimeric G proteins to induce GDP dissociation from the Gα subunit resulting in GTP binding, dissociation of Gα-GTP from Gβγ, and subsequent regulation of downstream effectors dependent on the specific G protein. These include regulation of adenylyl cyclase by G_s_ and G_i_, activation of phospholipase C-β (PLCβ) primarily by G_q_-family members, and activation of small G protein exchange factors by G_12/13_ (1, 2, 3). Ultimately, GPCRs are then further regulated by GPCR kinases and arrestins which function to modulate G protein signaling as well as GPCR trafficking (4).

Ca^2+^ mobilization is a well-known second messenger that can be induced by GPCR activation. The primary canonical pathway for GPCR-promoted Ca^2+^ mobilization involves Gα_q_-mediated activation of PLCβ, while Gβγ subunits from activated G proteins can also induce Ca^2+^ mobilization through PLCβ (5, 6, 7, 8). Indeed, recent studies support an independent role for Gα_q_ and Gβγ in PLCβ activation with Gα_q_ enhancing catalytic activity and Gβγ promoting membrane recruitment (9, 10). Activation promotes hydrolysis of phosphatidylinositol 4,5-bisphosphate which produces diacylglycerol to activate protein kinase C, and inositol trisphosphate (IP_3_) which binds to IP_3_ receptors localized at the endoplasmic reticulum to promote the release of Ca^2+^ (11).

Previous studies have thoroughly investigated GPCR-mediated Ca^2+^ mobilization through the β_2_-adrenergic receptor (β_2_AR). While several studies demonstrated the ability of endogenous or overexpressed β_2_AR to promote agonist-dependent Ca^2+^ mobilization in human embryonic kidney 293 (HEK293) cells (12, 13, 14), more recent studies started to dissect the mechanism of this process and reached different conclusions regarding β_2_AR-promoted Ca^2+^ mobilization in HEK293 cells (15, 16). Stallaert *et al.* found that β_2_AR-promoted Ca^2+^ mobilization in HEK293S cells was G_s_-dependent but cAMP-independent and involved release of ATP with subsequent activation of G_q_ and PLCβ through P2Y purinergic receptors (15). In contrast, Galaz-Montoya *et al.* found that β_2_AR-mediated Ca^2+^ mobilization in HEK293 cells involved PLCβ but did not involve cAMP, G_s_, or G_i_ (16). Thus, one study found a critical role for G_s_ while the other found no apparent role for G_s_. While the techniques used in these studies might have contributed to some of the differences in the conclusions, there are clear disparities in the potential role of G_s_ in β_2_AR-promoted Ca^2+^ mobilization.

Here, we sought to further characterize the role of G proteins in β_2_AR-promoted Ca^2+^ mobilization and expand on these studies to include additional G_s_-coupled receptors to try to dissect whether there are any common features that contribute to GPCR-mediated Ca^2+^ mobilization. Using Gα protein KO cells, G protein specific inhibitors, and endogenous GPCRs, we found significant differences in the signaling pathways that are used to mediate Ca^2+^ mobilization in HEK293 cells where the β_2_AR primarily uses G_q_, prostaglandin EP2 receptor (EP_2_R) uses G_s_ and G_i_, and prostaglandin EP4 receptor (EP_4_R) uses G_s_, G_i_, and G_q_.

## Results

### β-agonists selectively stimulate endogenous β_2_ARs to induce Ca^2+^ mobilization in HEK293 cells

**Figure 1:**
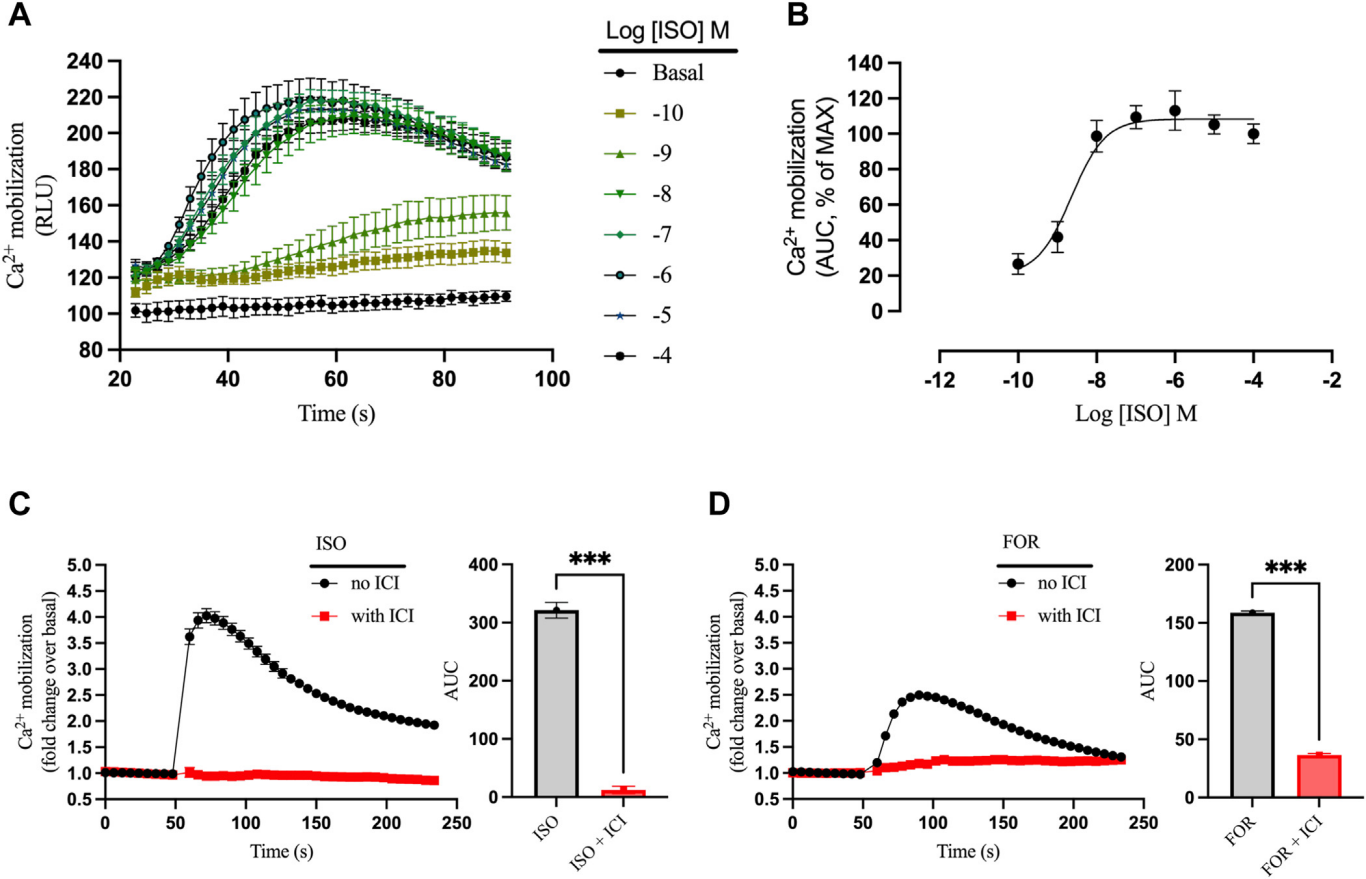
**Ca**^**2+**^**mobilization induced by β-agonists.** HEK293 cells endogenously expressing the β_2_AR were preincubated with the calcium dye FLUO-4 for 1 h; Ca^2+^ mobilization was then continuously monitored for up to 250 s after injection of β-agonist. *A*, Ca^2+^ mobilization upon stimulation with increasing concentrations of ISO was obtained using a FlexStation 3 Multi-Mode Microplate Reader that allows eight different channels to inject simultaneously. Basal represents cells stimulated only with assay buffer in the absence of agonist. Values are shown as relative light units (RLU). *B*, the area under the curve (AUC) of each concentration-dependent ISO-generated curve from panel *A* was extrapolated and plotted as a concentration/activity curve. Concentration/activity values are shown as % AUC compared to the maximal ISO concentration. ISO (*C*) and FOR (*D*) mediated calcium mobilization at 10 μM concentration in the presence/absence of 10 μM ICI were obtained using a CLARIOstar Plus Plate Reader. The AUC was calculated from each of the curves in panels *C* and *D* and plotted as mean ± SEM, n = 6. Statistical significance was assessed by *t* test with Welch’s correction, ∗∗∗*p* < 0.001. The ISO curve shown in panel *C* is the averaged signal from all ISO replicates (except Fig. 7) performed in WT cells throughout the study (n = 30). β_2_AR, β_2_-adrenergic receptor; FOR, formoterol; HEK293, human embryonic kidney 293; ICI, ICI-118551; ISO, isoproterenol.

Since HEK293 cells endogenously express a low level of β_2_ARs (17, 18), we initially evaluated the ability of the βAR-selective full agonist isoproterenol (ISO) to induce Ca^2+^ mobilization. ISO induced robust and transient Ca^2+^ mobilization in a concentration-dependent manner with the peak response reached at ∼25 s after agonist addition (Fig. 1*A*). The onset of the peak response right-shifted at lower ISO concentrations suggesting time-sensitive kinetics of Ca^2+^ mobilization associated with the different agonist concentrations. Moreover, the kinetics associated with higher ISO concentrations (10 nM–100 nM) were transient while profiles associated with lower concentrations (0.1–1 nM) were reduced but more sustained. Extrapolation of the area under the curve (AUC) to generate the related logistic concentration-activity curve showed an ISO potency of ∼2 nM and a maximum response starting at 100 nM (Fig. 1*B*). To verify that the observed effects with ISO were due to activation of the β_2_AR, we preincubated the cells with the β_2_AR selective antagonist ICI-118551 (ICI). ISO induced a robust and transient Ca^2+^ elevation that was completely inhibited by ICI (Fig. 1*C*). We also evaluated Ca^2+^ mobilization induced by formoterol (FOR), a β_2_AR selective agonist that is used clinically to treat chronic asthma. FOR also induced a rapid and transient Ca^2+^ mobilization that was effectively inhibited by ICI, although the peak intensity was only about half of that seen with ISO (Fig. 1*D*). It is worth noting that ICI did not interfere with global cellular Ca^2+^ mobilization since stimulation with ionomycin (IONO), an ionophore that elevates intracellular Ca^2+^ independently of GPCR activation, was not impacted by preincubation with ICI (Fig. S1). Taken together, these studies demonstrate that the endogenous β_2_AR in HEK293 cells effectively activates Ca^2+^ mobilization in an agonist-dependent manner.

### Ca^2+^ mobilization induced by the β_2_AR is primarily mediated by G_q_ in HEK293 cells

**Figure 2:**
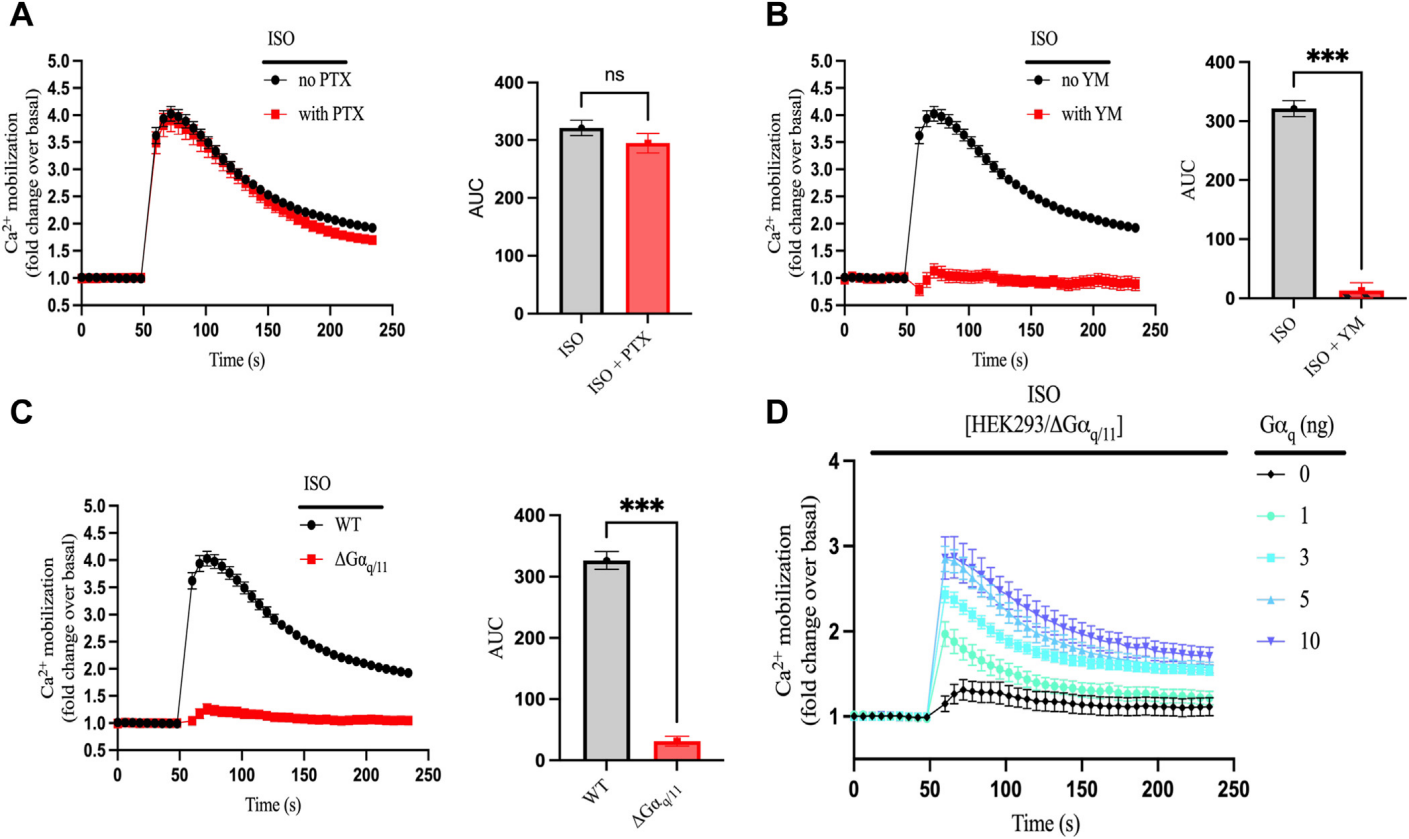
**β**_**2**_**AR-induced Ca**^**2+**^**mobilization is G**_**q**_**dependent and G**_**i**_**independent.***A*, HEK293 cells endogenously expressing the β_2_AR were preincubated with or without 100 ng/ml of the G_i_ inhibitor pertussis toxin (PTX) for 10 h with FLUO-4 added for the last hour. Intracellular Ca^2+^ mobilization was then continuously monitored after injection of 10 μM ISO. *B*, cells were preincubated with FLUO-4 for 1 h in the presence or absence of 1 μM of the G_q_ inhibitor YM-254890 (YM). Intracellular Ca^2+^ mobilization was then continuously monitored after injection of 10 μM ISO. *C*, comparison of intracellular Ca^2+^ mobilization between WT HEK293 cells and CRISPR/Cas9 Gα_q/11_ KO HEK293 (ΔGα_q/11_) cells upon stimulation with 10 μM ISO. *D*, ΔGα_q/11_ cells were transiently transfected with increasing concentration (0–10 ng/0.35 cm^2^ of cells) of a plasmid encoding the Gα_q_ protein. After 48 h, cells were preincubated with FLUO-4 for 1 h, and intracellular Ca^2+^ mobilization was continuously monitored after injection of 10 μM ISO. The AUC was calculated from each of the curves in panels (*A*–*C*) and plotted as histograms. All data are shown as fold change over basal and are plotted as mean ± SEM, n = 6. Statistical significance was assessed by *t* test with Welch’s correction, ∗∗∗*p* < 0.001, ns = not significant. The ISO curve shown in panels (*A*–*C*) is the averaged signal from all ISO replicates (except Fig. 7) performed in WT cells throughout the study (n = 30). β_2_AR, β_2_-adrenergic receptor; AUC, area under the curve; HEK293, human embryonic kidney 293; ISO, isoproterenol.

Ca^2+^ mobilization induced by GPCR activation is mediated by two main mechanisms, one that involves G_q_ and results in Gα_q_-mediated activation of PLCβ and another that involves G_i/o_ and results in Gβγ-mediated activation of PLCβ (19, 20). While the β_2_AR has been shown to activate G_i_ (21, 22), a direct role for G_q_ in β_2_AR signaling has not been established. To dissect the possible contribution of G_i_ in β_2_AR-mediated Ca^2+^ mobilization, we preincubated HEK293 cells for ten hours with or without pertussis toxin (PTX), a potent inhibitor of G_i_ family members. PTX treatment had no effect on ISO-promoted Ca^2+^ mobilization suggesting that it does not involve G_i_ (Fig. 2*A*). It is worth noting that preincubating the cells with PTX decreased IONO-mediated Ca^2+^ mobilization by ∼30% compared to untreated cells (Fig. S2*A*), although PTX treatment did not affect the peak response.

We also dissected the possible contribution of G_q_ in β_2_AR-mediated Ca^2+^ mobilization. To initially assess this, we preincubated HEK293 cells with or without the selective G_q_ inhibitor YM-254890 (YM) before ISO stimulation. ISO did not promote a significant calcium response in cells treated with YM (Fig. 2*B*), suggesting a primary role for G_q_ in β_2_AR-mediated Ca^2+^ mobilization. We did not observe any effect of YM on IONO-mediated intracellular Ca^2+^ mobilization (Fig. S2*B*). To corroborate the role of G_q_ in β_2_AR-mediated Ca^2+^ mobilization, we evaluated ISO effects in HEK293 cells that were knocked-out for Gα_q/11_ (ΔGα_q/11_) using CRISPR-Cas9. These cells did not generate any significant Ca^2+^ mobilization upon stimulation with ISO compared to WT cells (Fig. 2*C*). When G_q_ activity was restored in ΔGα_q/11_ cells by transfecting a plasmid encoding the Gα_q_ subunit, ISO-promoted Ca^2+^ mobilization was restored in a concentration dependent manner (Fig. 2*D*). Taken together, these studies demonstrate a major contribution of G_q_ in β_2_AR-mediated Ca^2+^ mobilization in HEK293 cells while excluding any involvement of G_i_.

### G_s_ activation contributes to the kinetics of β_2_AR-mediated Ca^2+^ mobilization

**Figure 3:**
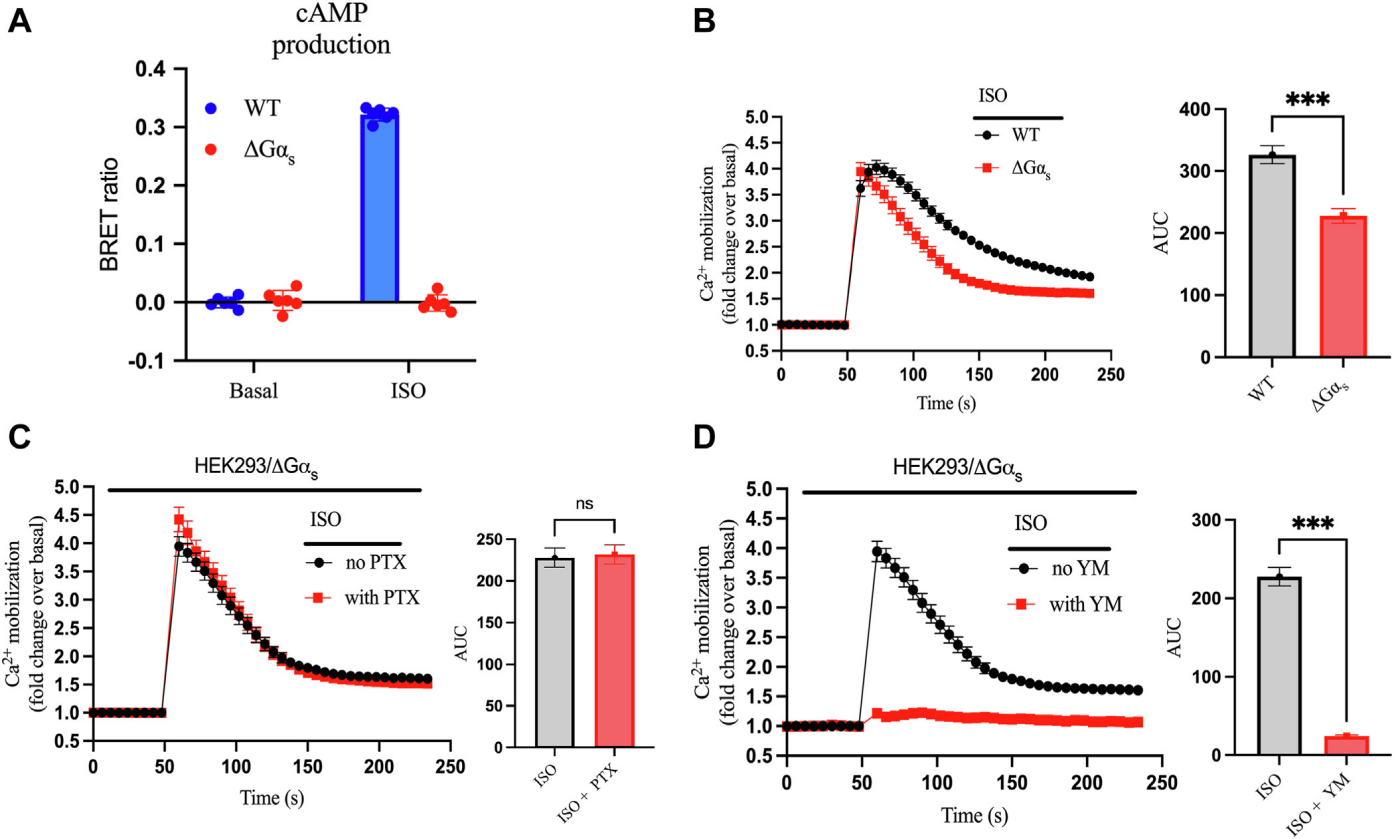
**G**_**s**_**activation minimally contributes to β**_**2**_**AR-induced Ca**^**2+**^**mobilization.***A*, WT cells or CRISPR/Cas9 Gα_s_ KO HEK293 (ΔGα_s_) cells were transiently transfected with the intramolecular BRET sensor CAMYEL to monitor cAMP production. After 48 h, cells were stimulated with or without 10 μM ISO, and the BRET signal was recorded. The data are expressed as the BRET ratio of acceptor/donor emission. *B*, comparison of intracellular Ca^2+^ mobilization between WT and ΔGα_s_ cells upon stimulation with 10 μM ISO. *C*, ΔGα_s_ cells were preincubated with or without 100 ng/ml PTX for 10 h with FLUO-4 added for the last hour. Intracellular Ca^2+^ mobilization was continuously monitored after injection of 10 μM ISO. *D*, cells were preincubated with FLUO-4 for 1 h in the presence or absence of 1 μM YM-254890. Intracellular Ca^2+^ mobilization was continuously monitored after injection of 10 μM ISO. The AUC was calculated from each of the curves in panels (*B*–*D*) and plotted as histograms. All data are shown as fold change over basal and are plotted as mean ± SEM, n = 6. Statistical significance was assessed by *t* test with Welch’s correction, ∗∗∗*p* < 0.001, ns = not significant. The curve generated by ISO in panel *B* is the average signal of all the replicates (except Fig. 7) in WT cells (n = 30) through all the experimental conditions presented in this study. β_2_AR, β_2_-adrenergic receptor; AUC, area under the curve; BRET, bioluminescence resonance energy transfer; HEK293, human embryonic kidney 293; ISO, isoproterenol; PTX, pertussis toxin.

While β_2_AR-mediated intracellular Ca^2+^ release has been previously correlated with G_s_ activation through either a cAMP-dependent or cAMP-independent mechanism in excitable cells (23, 24), a role for G_s_ in nonexcitable cells is less clear (15, 16). To probe the possible contribution of G_s_ signaling in Ca^2+^ dynamics upon β_2_AR activation, we stimulated HEK293 cells that were knocked-out for Gα_s/olf_ (ΔGα_s_) using CRISPR-Cas9. First, we confirmed that such cells are indeed devoid of G_s_ signaling by transiently transfecting the intramolecular bioluminescence resonance energy transfer (BRET) cAMP sensor CAMYEL (25). While WT HEK293 cells had robust ISO-mediated cAMP accumulation, ΔGα_s_ cells did not show any ISO-promoted cAMP confirming that ΔGα_s_ cells lack G_s_ signaling (Fig. 3*A*). Next, we compared ISO-mediated Ca^2+^ mobilization in WT *versus* ΔGα_s_ cells. The absence of G_s_ signaling had no impact on the peak β_2_AR-mediated Ca^2+^ mobilization; however, the kinetics of the response in ΔGα_s_ cells was more transient and the AUC was ∼30% lower compared to WT cells (Fig. 3*B*). IONO-mediated Ca^2+^ mobilization in ΔGα_s_ cells also displayed a different profile compared to WT cells, although the quantified AUC in ΔGα_s_ cells was modestly higher than in WT cells (Fig. S3*A*). Similar to what was observed in WT HEK293 cells, we did not see any effect of PTX preincubation on ISO-mediated Ca^2+^ mobilization in ΔGα_s_ cells (Fig. 3*C*), while preincubation with YM completely abrogated ISO-promoted Ca^2+^ release in ΔGα_s_ cells (Fig. 3*D*). Similar to WT cells, preincubation of ΔGα_s_ cells with PTX statistically decreased the IONO-mediated intracellular Ca^2+^ mobilization when compared to untreated cells (Fig. S3*B*) while YM had a modest effect (Fig. S3*C*). These data further corroborate the predominance of G_q_ signaling in initiating Ca^2+^ release upon β_2_AR activation and suggest a potential role of G_s_ in the kinetics of this process.

### The β_2_AR can recruit and activate G_q_ in BRET assays

**Figure 4:**
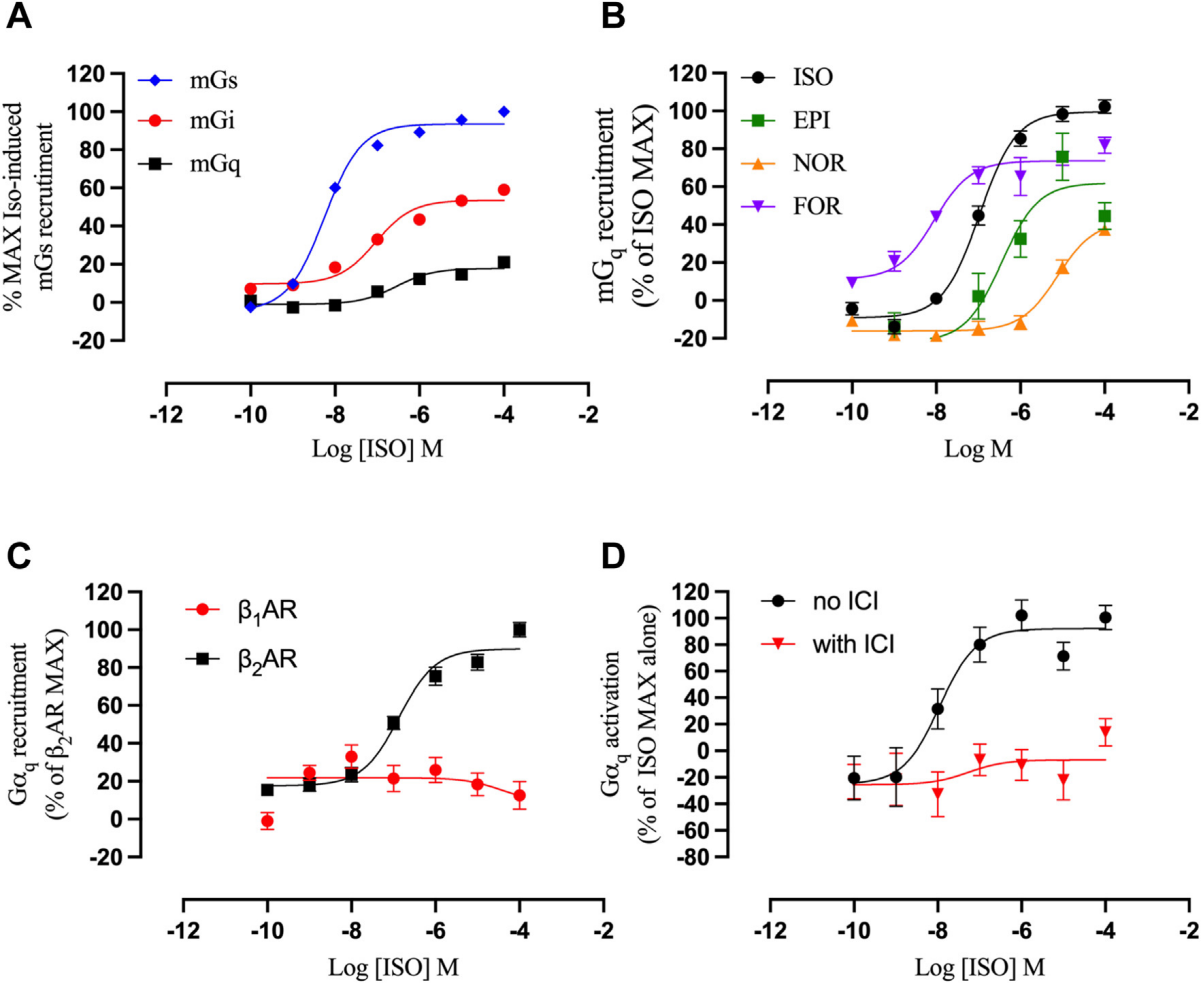
**BRET assay supports direct interaction between the β**_**2**_**AR and G**_**q**_. HEK293 cells were transiently transfected with BRET donor β_1_AR-RlucII, β_2_AR-RlucII, or GRK2-RH-NanoLuc and BRET acceptor NES-Venus-mGs, -mGsi, -mGsq, or Gα_q_-Venus. After 48 h, cells were stimulated with increasing concentrations of the respective agonist and the BRET signal was recorded after 20 min stimulation and plotted as concentration/activity curves. *A*, β_2_AR-mediated recruitment of mG proteins upon stimulation with increasing concentrations of the full agonist ISO. Data are shown as % of maximal ISO-induced mGs recruitment. *B*, β_2_AR-mediated recruitment of mGsq upon stimulation with increasing concentrations of several β-agonists (ISO), epinephrine (EPI), norepinephrine (NOR), and formoterol (FOR). Data are shown as % of maximal ISO-induced mGsq recruitment. *C*, β_1_AR- and β_2_AR-mediated recruitment of Gα_q_ upon stimulation with increasing concentrations of ISO. Data are shown as % of maximal ISO-induced Gα_q_ recruitment to the β_2_AR. *D*, activation of Gα_q_ by endogenous β_2_AR upon stimulation with increasing concentrations of ISO in the presence/absence of 10 μM ICI. Data are shown as % of maximal ISO-induced Gα_q_ activation in the absence of ICI. All normalized concentration/activity curves are plotted as mean ± SEM, n = 4. β_2_AR, β_2_-adrenergic receptor; BRET, bioluminescence resonance energy transfer; GRK2-RH, regulator of G protein signaling homology; HEK293, human embryonic kidney 293; ICI, ICI-118,551; ISO, isoproterenol; Rluc, Renilla luciferase.

Since our results support the involvement of G_q_ activation in β_2_AR-mediated Ca^2+^ mobilization in HEK293 cells, we hypothesized that direct interaction between activated β_2_AR and G_q_ might occur. To investigate this, we took advantage of a panel of BRET biosensors that infer protein-protein interaction by virtue of their proximal energy exchange. In the first set of experiments, we transiently transfected HEK293 cells with β_2_AR fused to the BRET donor Renilla luciferase (β_2_AR-Rluc) together with the mini G protein constructs mGs, mGsi, and mGsq. These are engineered Gα subunits fused to the BRET acceptor Venus that retain the ability to couple to activated GPCRs, therefore serving as informative pharmacological tools to assess GPCR-mediated signaling (26). ISO induced β_2_AR coupling to the different mG proteins with potencies and efficacies dependent on the nature of the particular Gα subunit (Fig. 4*A*). mGs coupling to the β_2_AR was the most potent and efficient upon ISO stimulation, followed by mGsi, which showed an efficacy of ∼60% compared to mGs. ISO was also able to promote mGsq recruitment to the β_2_AR with an efficacy of ∼20% compared to mGs. These data support the preferential coupling of the β_2_AR to G_s_ and the established secondary coupling to G_i_ while also providing evidence for coupling to G_q_. Due to the highly engineered nature of the mG constructs, we also evaluated the selectivity of these biosensors by determining mG coupling to the chemokine receptor CXCR4, which is canonically coupled to G_i_. HEK293 cells transiently transfected with CXCR4-Rluc and stimulated with the agonist CXCL12 showed robust coupling to mGsi but no coupling to mGs or mGsq, corroborating the selectivity of these constructs (Fig. S4). We also evaluated if the β_2_AR-mediated mGsq recruitment could be modulated by different β-agonists including the endogenous agonists epinephrine and norepinephrine, as well as FOR, and ISO. This analysis showed that ISO was the most efficacious in recruiting mGsq followed by FOR (∼80% of ISO), epinephrine (∼60%) and norepinephrine (∼40%) (Fig. 4*B*). Notably, FOR showed a better potency compared to ISO but reduced efficacy which correlates with the diminished Ca^2+^ mobilization induced by FOR compared to ISO (Fig. 1*D*).

To further gauge G_q_ coupling to the β_2_AR we tested the activity of a BRET biosensor composed of the holo-Gα_q_ subunit fused to the BRET acceptor Venus (Gα_q_-Venus) (27). HEK293 cells transiently transfected with β_2_AR-Rluc and Gα_q_-Venus induced Gα_q_ recruitment to the β_2_AR upon stimulation with increasing concentrations of ISO (Fig. 4*C*). The potency of ISO-mediated Gα_q_-Venus recruitment was comparable to that of mGsq recruitment further supporting β_2_AR-mediated coupling to G_q_. Importantly, we did not observe any ISO-mediated Gα_q_ recruitment when assessing the closely-related β_1_-adrenergic receptor (β_1_AR), supporting G protein subtype-selectivity for the β_1_AR. While the BRET biosensors described above are powerful tools to assess receptor/G protein interaction, they rely on overexpression of the receptor. To utilize a BRET assay with endogenous β_2_ARs, we took advantage of a recently described bimolecular BRET sensor system for G_q_ activation (28). This system involves the regulator of G protein signaling homology (RH) domain of GRK2 (GRK2^RH^) fused to NanoLuc (GRK2^RH^-Nluc) as BRET donor and Gα_q_-Venus as BRET acceptor. By virtue of the high affinity and specificity of GRK2^RH^ for GTP-bound Gα_q_, this system should be able to evaluate G_q_ activation by endogenous β_2_ARs. HEK293 cells transiently transfected with GRK2^RH^-Nluc and Gα_q_-Venus showed a concentration-dependent G_q_ activation upon stimulation with ISO (Fig. 4*D*). This ISO-mediated G_q_ activation was effectively inhibited by ICI demonstrating that it was β_2_AR specific. Collectively, these results strongly support agonist-promoted coupling of the β_2_AR to G_q_.

### The prostaglandin EP_2_ receptor promotes Ca^2+^ mobilization in a G_s_- and G_i_-dependent manner

**Figure 5:**
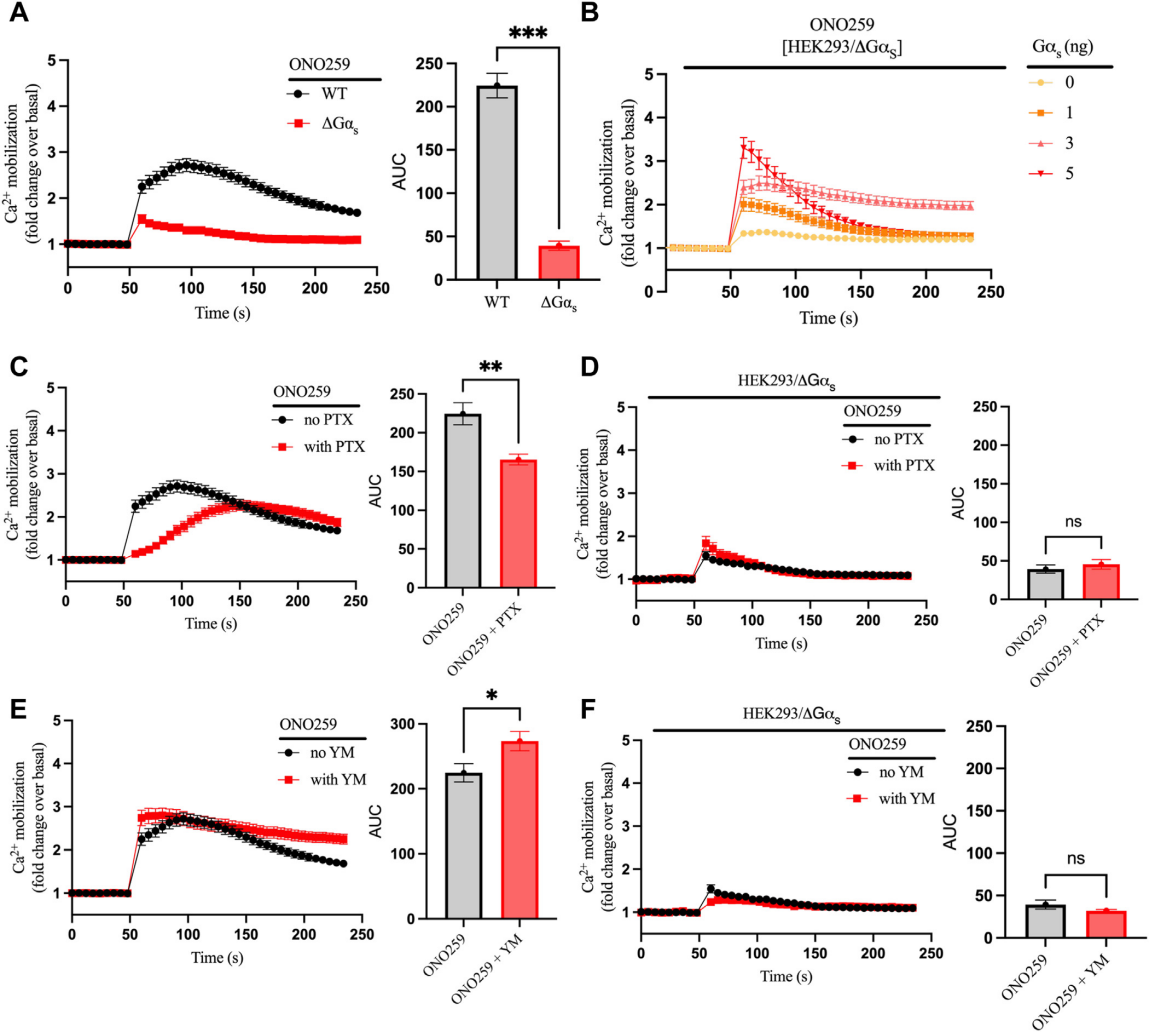
**EP**_**2**_**R-mediated Ca**^**2+**^**mobilization is G**_**s**_**and G**_**i**_**dependent and G**_**q**_**independent.***A*, comparison of intracellular Ca^2+^ mobilization between WT and ΔGα_s_ HEK293 cells upon stimulation with 1 μM ONO259. *B*, ΔGα_s_ cells were transiently transfected with increasing concentrations (0–5 ng/0.35 cm^2^ of cells) of a plasmid encoding the Gα_s_ protein. After 48 h, cells were preincubated with FLUO-4 for 1 h, and intracellular Ca^2+^ mobilization was continuously monitored after injection of 1 μM ONO259. (*C*) WT or (*D*) ΔGα_s_ cells endogenously expressing the EP_2_ receptor were preincubated with or without 100 ng/ml PTX for 10 h with FLUO-4 added for the last hour. Intracellular Ca^2+^ mobilization was continuously monitored after injection of 1 μM ONO259. (*E*) WT or (*F*) ΔGα_s_ HEK293 cells were preincubated with FLUO-4 for 1 h in the presence or absence of 1 μM YM-254890. Intracellular Ca^2+^ mobilization was continuously monitored after injection of 1 μM ONO259. The AUC was calculated from each of the curves in panels (*A*, *C*–*F*) and plotted as histograms. All curve data are shown as fold change over basal and are plotted as mean ± SEM, n = 6 except for panel (*B*) where n = 3. Statistical significance was assessed by *t* test with Welch’s correction, ∗*p* < 0.05, ∗∗*p* < 0.01, ∗∗∗*p* < 0.001, ns = not significant. The ONO259 curve shown in panels *B*, *C*, and *E* is the averaged signal from all ONO259 replicates (except Fig. 7) performed in WT cells throughout the study (n = 18). AUC, area under the curve; EP2R, prostaglandin EP2 receptor; HEK293, human embryonic kidney 293; PTX, pertussis toxin.

To assess if what we observed for β_2_AR-mediated calcium mobilization is recapitulated with other GPCRs canonically coupled to G_s_, we studied the EP_2_R and EP_4_R. These receptors are endogenously expressed in HEK293 cells (18) and EP_2_R selectively couples to G_s_ while EP_4_R couples primarily to G_s_ and secondarily to G_i_ (29, 30, 31, 32). In addition, highly selective synthetic agonists exist for these two receptors: ONO-AE1-259-01 (ONO259) for the EP_2_R and ONO-AE1-329 (ONO329) for EP_4_R (33). We initially focused on EP_2_R-mediated signaling, assessing the ability of ONO259 to induce calcium mobilization in WT and ΔGα_s_ HEK293 cells. ONO259 stimulation induced transient Ca^2+^ elevation with a peak at ∼50 s after addition in WT cells while the Ca^2+^ elevation was significantly attenuated in ΔGα_s_ cells (Fig. 5*A*). When G_s_ was restored in ΔGα_s_ cells by transfecting a plasmid encoding the Gα_s_ subunit, ONO259-induced Ca^2^⁺ mobilization was rescued in a concentration-dependent manner (Fig. 5*B*). We next dissected any contribution of G_i_ and G_q_ signaling in the EP_2_R-mediated Ca^2+^ mobilization by blocking the corresponding G proteins with PTX or YM treatment as done for the β_2_AR. Preincubation of WT cells with PTX before agonist stimulation delayed the onset of EP_2_R-mediated Ca^2+^ mobilization by ∼150 s and resulted in a significant decrease in the total Ca^2+^ response (Fig. 5*C*). Since ΔGα_s_ cells show a residual Ca^2+^ elevation upon ONO259 stimulation, we determined if this could arise from G_i_ activation by evaluating the Ca^2+^ response in ΔGα_s_ cells after PTX treatment (Fig. 5*D*). Interestingly, this showed no difference in the Ca^2+^ signal with or without PTX preincubation, suggesting a possible G protein-independent mechanism for the residual Ca^2+^ response. We also treated WT HEK293 cells with the G_q_ inhibitor YM for one hour before ONO259 stimulation. Inhibiting G_q_ modestly affected the kinetics of the Ca^2+^ response resulting in it being faster and more sustained than in the absence of YM and an overall ∼20% increase in the total Ca^2+^ response (Fig. 5*E*). Moreover, preincubation of ΔGα_s_ cells with YM followed by ONO259 stimulation had no effect confirming that G_q_ has no significant role in EP_2_R-mediated Ca^2+^ mobilization. Thus, Ca^2+^ mobilization induced by the activation of the endogenous EP_2_R in HEK293 cells primarily relies on G_s_ signaling with a role for G_i_ in the kinetics of the response and no contribution from G_q_.

### The prostaglandin EP_4_R promotes Ca^2+^ mobilization in a G_s_-, G_i_-, and G_q_-dependent manner

**Figure 6:**
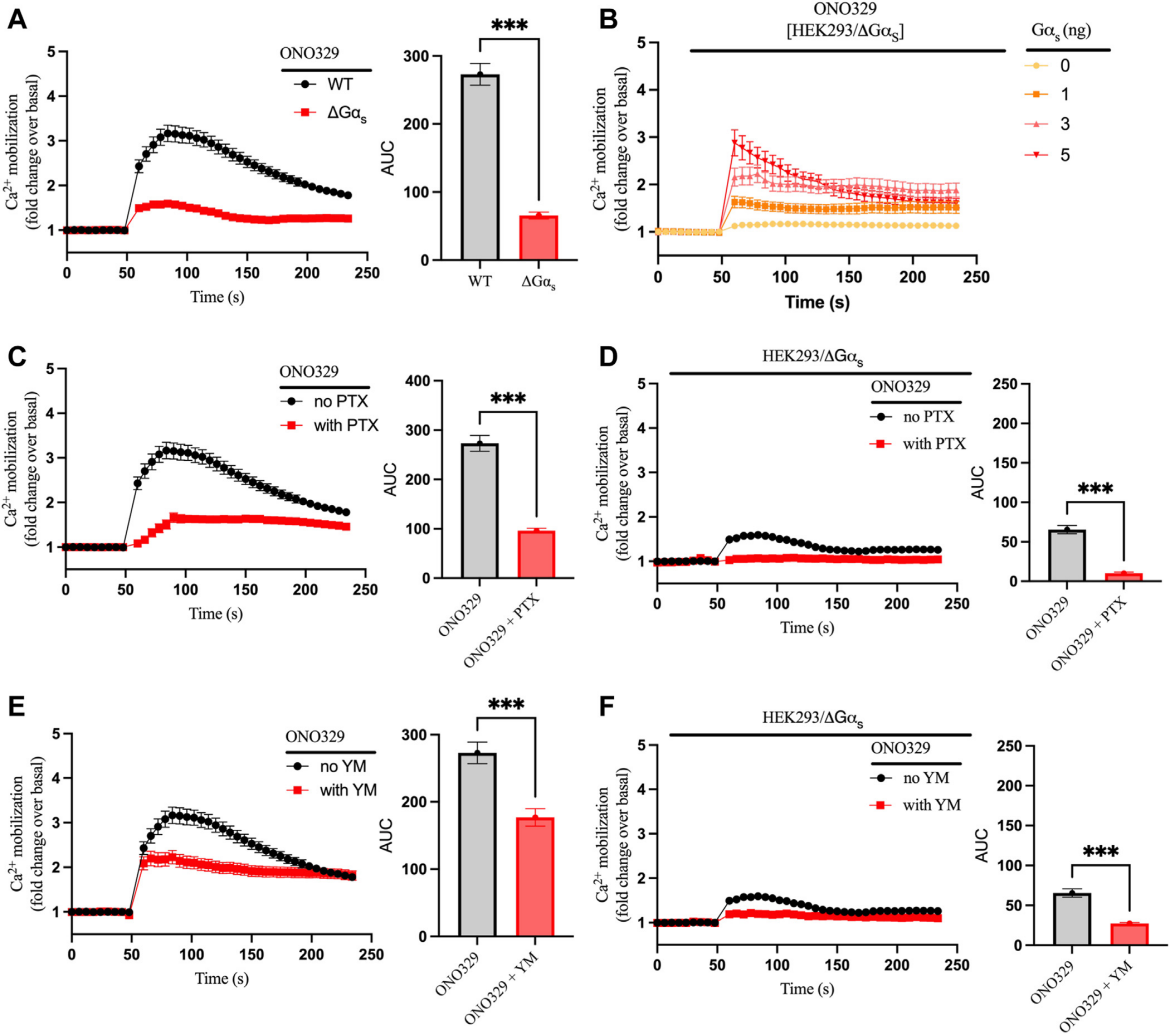
**EP**_**4**_**R-mediated Ca**^**2+**^**mobilization is G**_**s**_**, G**_**i**_**, and G**_**q**_**dependent.***A*, comparison of intracellular Ca^2+^ mobilization between WT and ΔGα_s_ HEK293 cells upon stimulation with 1 μM ONO329. *B*, ΔGα_s_ cells were transiently transfected with increasing concentrations (0–5 ng/0.35 cm^2^ of cells) of a plasmid encoding the Gα_s_ protein. After 48 h, cells were preincubated with FLUO-4 for 1 h, and intracellular Ca^2+^ mobilization was continuously monitored after injection of 1 μM ONO329. (*C*) WT or (*D*) ΔGα_s_ cells endogenously expressing the EP_4_ receptor were preincubated with or without 100 ng/ml PTX for 10 h with FLUO-4 added for the last hour. Intracellular Ca^2+^ mobilization was continuously monitored after injection of 1 μM ONO329. (*E*) WT or (*F*) ΔGα_s_ cells were preincubated with FLUO-4 for 1 h in the presence or absence of 1 μM YM-254890. Intracellular Ca^2+^ mobilization was continuously monitored after injection of 1 μM ONO329. The AUC was calculated from each of the curves in panels (*A*, *C*–*F*) and plotted as histograms. All curve data are shown as fold change over basal and are plotted as mean ± SEM, n = 6 except for panel (*B*) where n = 3. Statistical significance was assessed by *t* test with Welch’s correction, ∗∗∗*p* < 0.001. The ONO329 curve shown in panels *B*, *C*, and *E* is the averaged signal from all ONO259 replicates (except Fig. 7) performed in WT cells throughout the study (n = 18). AUC, area under the curve; EP4R, prostaglandin EP4 receptor; HEK293, human embryonic kidney 293; PTX, pertussis toxin.

We next explored EP_4_R-mediated signaling, which in addition to G_s_ can also couple to G_i_ (32). We initially investigated the ability of the EP_4_R selective agonist ONO329 to induce calcium mobilization in WT and ΔGα_s_ HEK293 cells. ONO329 induced transient Ca^2+^ elevation with a peak at ∼40 s after injection in WT cells while the Ca^2+^ mobilization was significantly attenuated in ΔGα_s_ cells (Fig. 6*A*). When G_s_ was restored in ΔGα_s_ cells by transfecting a plasmid encoding the Gα_s_ subunit, ONO329-induced Ca^2^⁺ mobilization was rescued in a concentration-dependent manner (Fig. 6*B*). Preincubation of WT cells with PTX for ten hours before agonist stimulation also significantly decreased EP_4_R-mediated Ca^2+^ mobilization (Fig. 6*C*), and this effect was also conserved in ΔGα_s_ cells (Fig. 6*D*), strongly supporting the concomitant role of G_i_ in the EP_4_R-mediated Ca^2+^ response. We also pretreated WT HEK293 cells with the G_q_ inhibitor YM prior to ONO329 stimulation (Fig. 6*E*). Similarly to G_i_, blocking G_q_ signaling reduced EP_4_R-mediated Ca^2+^ mobilization although to a lesser degree, suggesting that G_q_ also has a role in EP_4_R-mediated Ca^2+^ mobilization. However, the kinetics of the Ca^2+^ dynamics appeared to be different as it was faster and more sustained compared to that in the absence of YM. Finally, preincubation of ΔGα_s_ cells with YM followed by ONO329 stimulation showed a modest albeit significant decrease in Ca^2+^ mobilization when compared to untreated ΔGα_s_ cells, further supporting a role for G_q_ signaling in this process. In summary, Ca^2+^ mobilization induced by activation of the endogenous EP_4_R in HEK293 cells involves G_s_, G_i_, and G_q_.

### Contribution of extracellular Ca^2+^ and PLCβ to G_s_-coupled receptor-mediated Ca^2+^ mobilization

To further dissect the mechanism of calcium mobilization induced by G_s_-coupled receptors in nonexcitable cells, we examined the roles of extracellular calcium and PLCβ activity. Nonexcitable cells are believed to regulate extracellular calcium influx through mechanisms such as store-operated calcium entry (34, 35, 36) and transient receptor potential channels (37), which can be either GPCR-dependent or independent. To determine the contribution of these pathways to GPCR-mediated calcium mobilization, we compared calcium responses in the presence or absence of extracellular calcium. Preincubation of WT HEK293 cells in a calcium-free buffer led to a reduction in the calcium signal following ISO stimulation compared to cells in calcium-containing buffer (Fig. S5*A*), although this reduction was proportional to the decrease observed with IONO stimulation (Fig. S5*B*). A similar reduction was seen with the EP_2_R agonist ONO259 (Fig. S5*C*) and EP_4_R agonist ONO329 (Fig. S5*D*). Analysis of the AUC showed that all agonists induced similar calcium mobilization in the presence or absence of extracellular calcium when normalized to the IONO-derived AUC. These findings suggest that while extracellular calcium entry contributes to the overall calcium signal, it is not directly mediated by GPCRs and does not impact GPCR activity.

Given that the β₂AR mobilizes intracellular calcium *via* direct coupling to G_q_ while EP_2_R and EP_4_R primarily use G_s_, we next investigated the involvement of PLCβ enzymes in calcium mobilization. We first tried the reported PLCβ inhibitor U73122. Unexpectedly, preincubation of HEK293 cells with U73122 significantly increased basal calcium levels and led to a complete loss in the ability of IONO to elevate calcium (Fig. S6*A*). This suggests that U73122 may have saturated the calcium mobilization system. While U73122 is generally regarded as a broad-spectrum PLCβ inhibitor, one previous study reported that it activated certain PLCβ isozymes (38). These findings raise questions about the inhibitory properties of U73122 and underscore the need for caution when interpreting data obtained with U73122.

**Figure 7:**
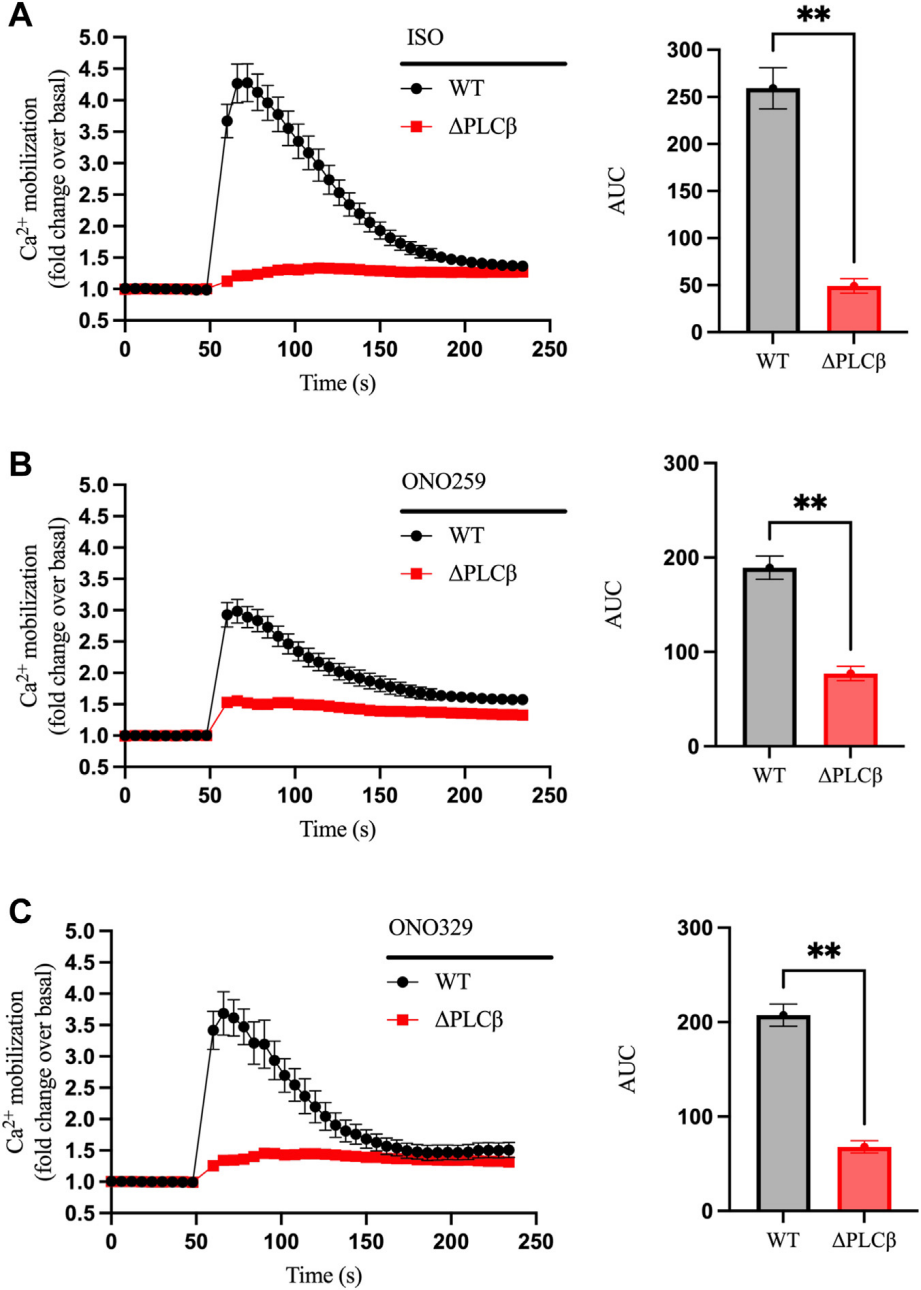
**PLCβ enzymes contribute to β**_**2**_**AR, EP**_**2**_**R, and EP**_**4**_**R-mediated Ca**^**2+**^**mobilization.** Comparison of intracellular Ca^2+^ mobilization between WT and ΔPLCβ HEK293 cells upon stimulation with (*A*) 10 μM ISO, (*B*) 1 μM ONO259, or (*C*) 1 μM ONO329. The AUC was calculated from each of the curves and plotted as histograms. All curve data are shown as fold change over basal and are plotted as mean ± SEM, n = 3. Statistical significance was assessed by *t* test with Welch’s correction, ∗∗*p* < 0.01. The ISO, ONO259, and ONO329 curves include the signal from replicates in the presence of extracellular Ca^2+^ performed in Fig. S5 (n = 6). β_2_AR, β_2_-adrenergic receptor; AUC, area under the curve; EP2R, prostaglandin EP2 receptor; EP4R, prostaglandin EP4 receptor; HEK293, human embryonic kidney 293; ISO, isoproterenol; PLCβ, phospholipase C-β.

To clarify the potential role of PLCβ in our system, we used a recently developed HEK293 cell line with CRISPR/Cas9-induced functional knockout of PLCβ1-4 isozymes (ΔPLCβ) (39). In ΔPLCβ cells, stimulation with ISO (Fig. 7*A*), ONO259 (Fig. 7*B*), and ONO329 (Fig. 7*C*) resulted in a significant reduction in receptor-mediated calcium mobilization compared to WT cells. In contrast, we did not observe any significant difference in IONO-mediated calcium elevation in WT *versus* ΔPLCβ cells (Fig. S6*B*). Taken together, these studies identify an important role for PLCβ in G_q_-dependent (β_2_AR) and G_s_-dependent (EP_2_R and EP_4_R) calcium mobilization.

## Discussion

In this study, we investigated the ability of several G_s_-coupled GPCRs to promote agonist-dependent Ca^2+^ mobilization in HEK293 cells. Using pharmacological and genetic approaches, we find that β_2_AR activated Ca^2+^ mobilization is predominantly mediated by G_q_ while proximity-based BRET analysis supports β-agonist-dependent coupling between the β_2_AR and G_q_. To generalize what was observed for the β_2_AR, we find that the G_s_-coupled EP_2_R mobilizes calcium in a G_s_- and G_i_-dependent manner while the closely related EP_4_R modulates calcium in a G_s_-, G_i_-, and G_q_-dependent manner. Importantly, for all the GPCRs analyzed in this study, calcium mobilization was consistently linked to the activation of PLCβ enzymes, regardless of the differences in upstream G-protein network activation. Therefore, our studies suggest that calcium mobilization induced by GPCRs that primarily couple to G_s_ is largely dependent on the coupling promiscuity of the receptor rather than being governed only by the nature of the effector involved. Indeed, multiple G proteins regulated calcium signaling across activation of three different G_s_-coupled GPCRs.

Classically, GPCRs modulate intracellular calcium by inducing either Gα_q_ activation or G_i_-derived Gβγ activation of PLCβ (5). Yet, Ca^2+^ elevation occurring from activation of G_s_-coupled GPCRs in nonexcitable cells is also common, although mechanistic insight on how this occurs is lacking (40). The β_2_AR represents a good model to study since it canonically couples to G_s_ and secondarily to G_i_ and it can induce intracellular Ca^2+^ mobilization in some nonexcitable cells. In this context, previous studies correlated β_2_AR-induced Ca^2+^ mobilization with cAMP-dependent activation of Epac leading to a PLCβε-mediated increase in intracellular Ca^2+^ in HEK293 cells (12). Although in the present study we find that G_s_ is not required for β_2_AR-mediated calcium mobilization, we cannot exclude that PLCβε might contribute to the differences in Ca^2+^ kinetics that we observed in WT *versus* ΔGα_s_ cells. In this regard, it is worth noting that GPCR-dependent Ca^2+^ mobilization only marks the initial spike of the induced calcium elevation prompted by the fast opening of the Ca^2+^ ion channels present in the endoplasmic reticulum. The subsequent rapid decay of the calcium signal denotes the equally rapid reuptake of Ca^2+^ ions driven by several GPCR-independent mechanisms, which, among others, involve calcium-binding proteins and the sarco/endoplasmic reticulum Ca^2+^-ATPase (41, 42, 43). Since HEK293 cells that were knocked out for G proteins have been shown to promote cellular rewiring (44), the differences in the later kinetics of Ca^2+^ mobilization that we observed might also be governed by the different genetic backgrounds between WT and ΔGα_s_ cells that lead to diverse regulation of those GPCR-independent mechanisms that rapidly remove Ca^2+^ ions from the cytosol. A deeper analysis of such kinetic mechanisms would be required to dissect this phenomenon.

Studies by Stallaert *et al.* reported that the β_2_AR-mediated Ca^2+^ mobilization in HEK293 cells was dependent on G_s_ activation but independent of cAMP accumulation (15). The authors proposed a mechanism that involves the β_2_AR-induced and G_s_-mediated extracellular release of ATP, resulting in activation of G_q_-coupled P2Y purinergic receptors and leading to an increase in IP_3_-dependent intracellular Ca^2+^ mobilization, hence establishing signaling crosstalk between the β_2_AR and P2Y receptor. Although transactivation between the adrenergic and purinergic receptors has been described previously (45), this does not appear to play a significant role in β_2_AR-mediated calcium mobilization in HEK293 cells in our studies. Indeed, ISO stimulation of cells depleted of G_s_ induced robust Ca^2+^ mobilization comparable to that of WT cells. In this context, there are some notable experimental differences between our study and that of Stallaert *et al.* that may have contributed to the different responses. First, most of the experiments evaluating Ca^2+^ mobilization in Stallaert *et al.* used HEK293 cells overexpressing the β_2_AR while we used HEK293 cells with endogenous β_2_AR. While overexpression of a specific component of a signaling pathway may help stabilize and enhance the signal during detection, this can also change normal cellular networks and lead to altered cellular responses (46, 47, 48). In addition, while both studies used the same ΔGα_s_ cell line developed by the Inoue laboratory, it is possible that subsequent cell culture led to differences in the clones. Such differences have previously been reported in WT HEK293 cells (49, 50). Finally, the Ca^2+^ detection methods used in the two studies might have contributed to differences. While we used the established fluorescent Ca^2+^ dye Fura-4 which is cell-permeable and does not require any modification to the cells, Stallaert *et al.* detected Ca^2+^ by transfecting the cells with the mCherry-obelin- or GFP-obelin-based Ca^2+^ sensitive BRET biosensors. While we have no clear explanation for the differences in the findings, perhaps a combination of β_2_AR expression level, clonal differences in the cell lines, and Ca^2+^ detection methods might have contributed to the observed differences.

In another article interrogating the molecular mechanism behind the β_2_AR-mediated Ca^2+^ mobilization, Galaz-Montoya *et al.* show that β_2_AR-mediated Ca^2+^ mobilization arises from the activation of the PLC/IP_3_ pathway in HEK293 cells and use PTX and protein kinase A inhibitors to conclude that Ca^2+^ mobilization is independent of G_s_ or G_i_ activation (16). Thus, this study supports the extraneity of G_s_ and G_i_ signaling to β_2_AR-mediated Ca^2+^ mobilization. The authors also show that activated β_2_AR was unable to induce a membrane potential similar to that induced by the M3-muscarinic receptor (M3R). Since the authors had previously shown that the M3R-induced membrane potential is mediated by G_q_ (51), they concluded that the β_2_AR does not activate G_q_. This is in contrast with our findings, which clearly show that β_2_AR-mediated Ca^2+^ mobilization is G_q_-dependent. Notably, the possibility that the β_2_AR can directly couple to G_q_ is an exciting finding that could lead to a re-consideration of the β_2_AR coupling promiscuity. It is worth noting that previous reports failed to establish a β_2_AR-G_q_ association in studies involving large-scale GPCR-G-protein association screening (52, 53, 54). These differences might be explained by the very low efficiency that G_q_ shows for the β_2_AR compared to G_s_ or G_i_. Indeed, ISO stimulation recruited mGsq at only ∼20% of the mGs recruitment denoting a weak association upon β_2_AR activation. This is also in accordance with the first published report of the mG BRET sensors where it was shown that the β_2_AR weakly recruits mGsq (26). This weak association might be one reason why large screening studies did not detect G_q_ coupling to the β_2_AR as well as why activation of the β_2_AR did not induce an appreciable membrane potential compared to the G_q_-coupled M3R. Indeed, β_2_AR has been reported to directly couple to G_q_ in a study using overexpression systems in which the Gα_q_ protein was fused to the β_2_AR (55). Bringing in proximity two naturally interacting proteins greatly enhances their affinity, permitting the detection of their association even when they interact weakly. Further in-depth studies are needed to establish if G_q_ can couple to the β_2_AR in other cellular systems and whether such coupling has a physiological role.

Another interesting finding of our study reveals that different GPCRs that primarily couple to G_s_ can use different G protein networks to modulate intracellular Ca^2+^ mobilization. We studied the EP_2_ and EP_4_ receptors that are endogenously expressed in HEK293 cells and canonically coupled to G_s_ and G_s_/G_i_, respectively (29, 30, 31, 32). Unlike the β_2_AR, G_s_ activation was required to initiate EP_2_ and EP_4_ receptor-mediated Ca^2+^ mobilization. This is in line with previous reports identifying a role for G_s_ in cAMP-dependent and PKA-dependent Ca^2+^ mobilization upon EP_2_R or EP_4_R activation, respectively (56, 57). Interestingly, activation of Ca^2+^ mobilization by EP_2_R also involved G_i_ while activation of EP_4_R involved G_i_ and G_q_. To the best of our knowledge, this is the first report showing that EP_2_R signaling can be modulated by G_i_ in addition to G_s_ and that EP_4_R signaling can be modulated by G_q_ in addition to G_s_ and G_i_. A more extensive analysis is needed to further characterize the G-protein promiscuity of these two receptors. The evidence that EP_2/4_ receptor-mediated Ca^2+^ mobilization can be partially modulated by G_i_ or a combination of G_i_ and G_q_ activity is in accordance with recent reports that described two independent molecular mechanisms for G_i_-dissociated Gβγ-dependent and G_q_-dependent PLCβ activation. Indeed, Gβγ can activate PLCβ by increasing its membrane recruitment while Gα_q_ enhances catalysis (9, 10). Although these mechanisms can operate independently, they can also cooperate to increase PLCβ activity as a product of the two separate contributions.

Another proposed mechanism links G_i_-mediated activation of PLCβ to the hierarchical upstream activation of G_q_. In this context, the activation of PLCβ by G_i_-dissociated Gβγ cannot occur in the absence of the Gα_q_-mediated release of an autoinhibitory region in the catalytic site of PLCβ, therefore setting Gα_q_ as a necessary master switch for Gβγ-mediated activation of PLCβ (58). A recent study expanded on this hierarchical G_q_ activation mechanism to explain how G_s_-coupled receptor activation leads to intracellular Ca^2+^ elevation (39). They reported that Gβγ subunits released during G_s_ activation can stimulate specific PLCβ isozymes, although G_q_ priming by ATP or carbachol was found to be necessary for this process. While a role for G_s_-derived Gβγ in Ca^2+^ mobilization is an important observation (39), our studies did not require G_q_ priming for G_s_-coupled receptor-mediated Ca^2+^ mobilization for the β_2_AR, EP_2_R, or EP_4_R. Indeed, our studies as well as previous studies on β_2_AR-mediated calcium mobilization in HEK293 cells by Stallaert *et al.* (15) and Galaz-Montoya *et al.* (16) did not require G_q_ priming. Perhaps some of the differences in these studies can be attributed to clonal heterogeneity of HEK293 cells grown in different labs. A striking example of this was reported by the Lefkowitz lab who observed that the PTX-dependence of β_2_AR-mediated activation of ERK was highly variable in HEK293 cells grown by different individuals within the lab from completely PTX sensitive to completely insensitive (49). Thus, while Gβγ subunits from G_s_ appear to play an important role in calcium mobilization (39), a requirement for Gα_q_ in Gβγ-mediated calcium mobilization is less clear.

In conclusion, this study highlights a previously unexplored intertwined network of G protein contributions to calcium signaling driven by the activation of several G_s_-coupled GPCRs. This might pose the need for a more careful reevaluation of receptor coupling promiscuity correlated with specific signaling pathways, such as Ca^2+^ signaling. Ultimately, this study expounded the evidence of β_2_AR coupling to G_q_, EP_2_R to G_i_ and EP_4_R to G_q_ in addition to the canonical G proteins associated with these receptors.

### Limitations of study

Although we infer protein-protein interaction from the BRET analysis to support direct coupling of G_q_ to the activated β_2_AR, we acknowledge this as a potential limitation of the study since BRET analysis is a measure of proximity between two proteins tagged with a BRET donor and acceptor that are close enough to generate a signal (<10 nm). While we studied endogenous GPCRs in HEK293 cells, future studies should evaluate these signaling pathways in primary cells and establish whether these pathways have a physiological role.

## Experimental procedures

### Materials

All β-agonists, as well as the β_2_AR selective antagonist ICI were purchased from Sigma-Aldrich. EP receptor agonists ONO259 and ONO329 were kindly provided by Dr Raymond B. Penn (Thomas Jefferson University). Compounds were dissolved in water with 1 mM ascorbic acid or in dimethyl sulfoxide (Sigma-Aldrich), stored as stock solutions at −20 °C, and then diluted in Dulbecco’s phosphate buffered saline (Corning Inc) for analysis. Costar ninety-six-well black, clear-bottom microplates for Ca^2+^ assays were from Corning. Fluo-4 NW Calcium Assay Kit was from Invitrogen. PTX was from Enzo Life Sciences, while YM was from Focus Biomolecules or Wako Pure Chemical Industries. U73122 was from Bio-Techne Tocris. Ninety-six-well, bottom-opaque, white microplates for BRET assays were from PerkinElmer. Renilla Luciferase substrate coelenterazine H was from Cayman Chemical Company. WT HEK293 cells were purchased from the American Type Culture Collection. HEK293/ΔGα_s/olf_, HEK293/ΔGα_q/11_, and HEK293/ΔPLCβ cells were generated and characterized previously (15, 39, 59).

### Cell culture and transfection

WT HEK293 cells, ΔGα_q/11_ (Δ*GNAQ*/*GNA11*) cells, ΔGα_s/olf_ (Δ*GNAS*/*GNAL*) cells and ΔPLCβ (Δ*PLCB1*/*PLCB2*/*PLCB3*/*PLCB4*) cells were maintained in minimum essential medium Eagle with Earle’s salts and L-glutamine (Corning Inc) supplemented with 10% fetal bovine serum (Corning Inc), 100 U/ml penicillin and 0.1 mg/ml streptomycin (P/S) (Gibco). Cells were incubated at 37 °C in a humidified incubator with 5% CO_2_. Transient transfections were performed in suspension in a 96-well plate using Metafectene PRO (Biontex) following the manufacturer’s protocol. Briefly, two solutions were prepared and incubated separately for 5 min. One solution contained plasmid DNAs encoding for BRET acceptor and donor diluted in 25 μl per well of Dulbecco’s modified Eagle’s medium (DMEM, Gibco) containing 4.5 g/l D-glucose without phenol red while the other solution contained 0.5 μl per well of Metafectene Pro diluted in 25 μl per well of DMEM containing 4.5 g/l D-glucose without phenol red. For the G_q_ and G_s_ rescue experiments, ΔGα_q/11_ or ΔGα_s_ cells were transiently transfected with increasing concentrations (0–10 ng/0.35 cm^2^ of cells) of a plasmid encoding the Gα_q_ or Gα_s_ protein. After the first incubation, the solutions were mixed, incubated for 20 min, added to 80,000 cells per well diluted in a final volume of 200 μl per well of DMEM without phenol red containing 4.5 g/l D-glucose, 10% fetal bovine serum, and P/S, and dispensed in 96-well, bottom-opaque, white microplates.

### Calcium mobilization assay

We used the Fluo-4 NW Calcium Assay Kit (Invitrogen) to investigate calcium mobilization, and the experiments were performed according to the manufacturer’s protocol. Briefly, the day before the calcium assay, 80,000 cells/well in a final volume of 200 μl/well of complete DMEM without phenol red were plated in 96 well, clear-bottom, black plates, and incubated overnight. The following day, cell media were aspirated and replaced with fresh media with or without 100 ng/ml of PTX and incubated for 10 h according to the experimental set-up. One hour before reading, the media were removed and replaced with 100 μl/well of assay buffer or Hanks' balanced salt solution without Ca^2+^ and Mg^2+^ in the presence of 20 mM Hepes buffer containing 1× Fluo-4 NW calcium-dye mix and 2.5 mM probenecid in the presence/absence of 10 μM of ICI, 10 μM YM, or 10 μM U73122 according to the experimental setup, followed by 1 h incubation of the cells in the dark. Then, 20 μl/well of agonist or IONO diluted in the appropriate buffer were injected into each well to achieve the desired final concentration, and the signal was immediately recorded and monitored over time (up to 90 or 280 s) with a FlexStation 3 Multi-Mode Microplate Reader (Molecular Devices) or a CLARIOstar Plus Plate Reader (BMG labtech). The FlexStation system has eight channels and uses a monochromator for detection while the CLARIOstar system has only one channel but has increased sensitivity due to the specific set of wave-length-dedicated filters. Before agonist injection, a basal signal was recorded for up to 50 s, which served to normalize the signal across experiments.

### Bioluminescence resonance energy transfer assay

In order to measure the real-time cAMP response, WT and ΔGα_s_ HEK293 cells with endogenous β_2_AR were transfected with the BRET-based intramolecular cAMP sensor CAMYEL (both the donor and the acceptor are fused to the cAMP binding domain of exchange protein directly activated by cAMP) that upon cAMP binding undergoes a conformational change resulting in a change in the BRET signal (25). To evaluate mini-G protein recruitment, WT HEK293 cells were transiently transfected with β_2_AR or CXCR4 C terminally fused with donor RlucII (β_2_AR-RlucII or CXCR4-RlucII) along with acceptor NES-Venus-mGs, NES-Venus-mGsi, or NES-Venus-mGsq (26). For the assessment of Gα_q_ recruitment, WT HEK293 cells were transiently cotransfected with plasmids for donor β_2_AR-RlucII or β_1_AR-RlucII and acceptor Gα_q_-Venus (27). To measure Gα_q_ activation with endogenous β_2_AR, WT HEK293 cells endogenously expressing the β_2_AR were transiently transfected with donor GRK2^RH^-Nluc and acceptor Gα_q_-Venus. Forty-eight hours after transfection, media were removed and the cells were stimulated for 20 min with increasing concentrations of β-agonist or CXCL12 (at the concentration reported in the figure legends) in the presence of 5 μM coelenterazine H diluted with PBS to a final volume of 50 μl per well. Signals at 395 nm (donor) and 510 nm (acceptor) were recorded in an Infinite F500 plate reader (Tecan).

### Statistical analysis and data representation

All generated curves and statistical analyses were done using GraphPad Prism version 10.2.0 (GraphPad Software; https://www.graphpad.com/features). All the calcium kinetic curves are displayed as fold change over basal (except for Figs. 1*A* and S6*A*, which are expressed as relative light units). To do this, a single value was calculated by averaging the basal signal before agonist injection from 0 to 50 s for each replicate. Then the ratio of each value (comprising all points from 0–280 s) over the average basal value was calculated and plotted as a kinetic curve over time. The AUC of each kinetic curve describing calcium dynamics was extrapolated by using the function “Area Under Curve” in GraphPad Prism, where the baseline was set to 1, and only the points above the baseline were considered. The concentration-activity curves were extrapolated by using the function “log (agonist) *versus* response (three parameters)” in GraphPad Prism. For normalization, we first subtracted the basal signal (wells stimulated with PBS in the absence of ligand) from each stimulated well, and the values of all replicates were then divided by the mean of the reference value considered in each experiment and multiplied by 100 for any given read-out. Data are shown as the mean ± SEM from at least three independent experiments (each figure legend states the specific number of replicates). In particular, the curve generated by ISO, ONO259, and ONO329 in WT HEK293 cells shown is the averaged signal of all replicates performed in WT cells presented in this study (n = 30 for ISO; n = 18 for ONO259 and n = 18 for ONO259). Statistical significance was assessed by *t* test with Welch’s correction, ∗*p* < 0.05; ∗∗*p* < 0.01; and ∗∗∗*p* < 0.001.

## Data availability

All study data are included in the article and SI Appendix.

## Supporting information

This article contains supporting information.

## Conflict of interest

The authors declare that they have no conflicts of interest with the contents of this article.
