# Supplementary material for: Diverse pathways in GPCR-mediated activation of Ca2+ mobilization in HEK293 cells
Source: J Biol Chem. 2024 Oct 10;300(11):107882. doi: 10.1016/j.jbc.2024.107882 (PMC11570840; doi:10.1016/j.jbc.2024.107882)
Supplement: Supporting Information [file mmc1.pdf]

Diverse pathways in GPCR-mediated activation of Ca^{2+} mobilization in HEK293 cells

Francesco De Pascali, Asuka Inoue and Jeffrey L. Benovic

Supplementary figures

Figure S1. *IONO-mediated Ca^{2+} mobilization with or without preincubation with ICI.* HEK293 cells were preincubated with the calcium dye FLUO-4 for 1 h in the presence/absence of 10 μM of the β_2AR-selective antagonist ICI-118,551 (ICI). Then, intracellular Ca^{2+} mobilization was continuously monitored after injection of 1 μM ionomycin (IONO). Ionomycin is an ionophore that elevates intracellular Ca^{2+} independently of GPCR activation. Data are represented as fold change over basal. The AUC was calculated and plotted as histograms. All values were plotted as mean ± SEM, n=6. Statistical significance was assessed by t-test with Welch's correction, ns = not statistically significant.

Figure S2. *IONO-mediated Ca^{2+} mobilization with or without preincubation with PTX or YM.* **A)** HEK293 cells were preincubated with or without 100 ng/ml of the Gi inhibitor pertussis toxin (PTX) for 10 h. At the 9th hour of PTX incubation, the FLUO-4 calcium dye was added, and the cells were incubated for 1 h. Then, intracellular Ca^{2+} mobilization was continuously monitored after injection of 1 μM IONO. **B)** Cells were preincubated with FLUO-4 for 1 h in the presence/absence of 1 μM of the Gq inhibitor YM-254890 (YM). Intracellular Ca^{2+} mobilization was continuously monitored after injection of 1 μM of IONO. The AUC was calculated and plotted as histograms. All curve data are represented as fold change over basal. Values were plotted as mean ± SEM, n=6. Statistical significance was assessed by t-test with Welch's correction, ***p < 0.001, ns = not statistically significant.

Figure S3. *IONO-mediated Ca^{2+} mobilization in HEK293/$\Delta G\alpha_s$ cells.* **A)** Comparison of intracellular Ca^{2+} mobilization between WT and $\Delta G\alpha_s$ cells upon stimulation with 1 μM IONO. **B)** $\Delta G\alpha_s$ cells were preincubated with or without 100 ng/ml of the Gi inhibitor pertussis toxin (PTX) for 10 h. At the 9th hour of PTX incubation, the FLUO-4 calcium dye was added, and the cells were incubated for 1 h. Then, intracellular Ca^{2+} mobilization was continuously monitored after injection of 1 μM of IONO. **C)** Cells were preincubated with FLUO-4 for 1 h in the presence/absence of 1 μM of the Gq inhibitor YM-254890 (YM). Intracellular Ca^{2+} mobilization was continuously monitored after injection of 1 μM of IONO. The AUC was calculated from each of the curves and plotted as histograms. All curve data are represented as fold change over basal. Values were plotted as mean ± SEM, n=6. Statistical significance was assessed by t-test with Welch's correction, ***p < 0.001.

Figure S4. *Specificity of the mini-G protein constructs.* HEK 293 cells were transiently transfected with BRET donor CXCR4-Rluc and BRET acceptor NES-Venus-mGs, -mGi, or -mGq. After 48 h, cells were stimulated with increasing concentrations (10^{-7} – 10^{-13} M) of CXCL12, and the BRET signal was recorded after 20 min stimulation and plotted as concentration/activity curves. Data are shown as % of maximal CXCL12-induced mGi recruitment. All normalized concentration/activity curves are plotted as mean ± SEM, n=4.

Figure S5. *Agonist-promoted Ca^{2+} mobilization with or without extracellular Ca^{2+}.* HEK293 cells were preincubated with the calcium dye FLUO-4 diluted in either assay buffer (containing Ca^{2+})

or HBSS without Ca^{2+} and Mg^{2+} in the presence of 20 mM HEPES buffer. Intracellular Ca^{2+} mobilization was then continuously monitored after injection of **A)** 10 μM ISO, **B)** 1 μM IONO, **C)** 1 μM ONO259 or **D)** 1 μM ONO329. Data are represented as fold change over basal. The AUC from cells stimulated in the **E)** presence or **F)** absence of extracellular calcium ($[Ca^{2+}]_{ex}$) was calculated and plotted as histograms. All values are plotted as mean ± SEM, n=3.

Figure S6. *Effects of U73122 and PLCβ knockout on IONO-mediated* Ca^{2+} *mobilization.* **A)** HEK293 cells were preincubated with the calcium dye FLUO-4 with or without 10 μM of the PLCβ inhibitor U73122. Intracellular Ca^{2+} mobilization was then continuously monitored after injection of 1 μM IONO. Data are represented as relative light units (RLU) and all values are plotted as mean ± SEM, n=3. ***B)*** Comparison of intracellular Ca^{2+} mobilization between WT and ΔPLCβ HEK293 cells upon stimulation with 1 μM IONO. The AUC was calculated from each of the curves and plotted as histograms. All curve data are represented as fold change over basal. Values are plotted as mean ± SEM, n=3. Statistical significance was assessed by t-test with Welch's correction, ns=not significant.

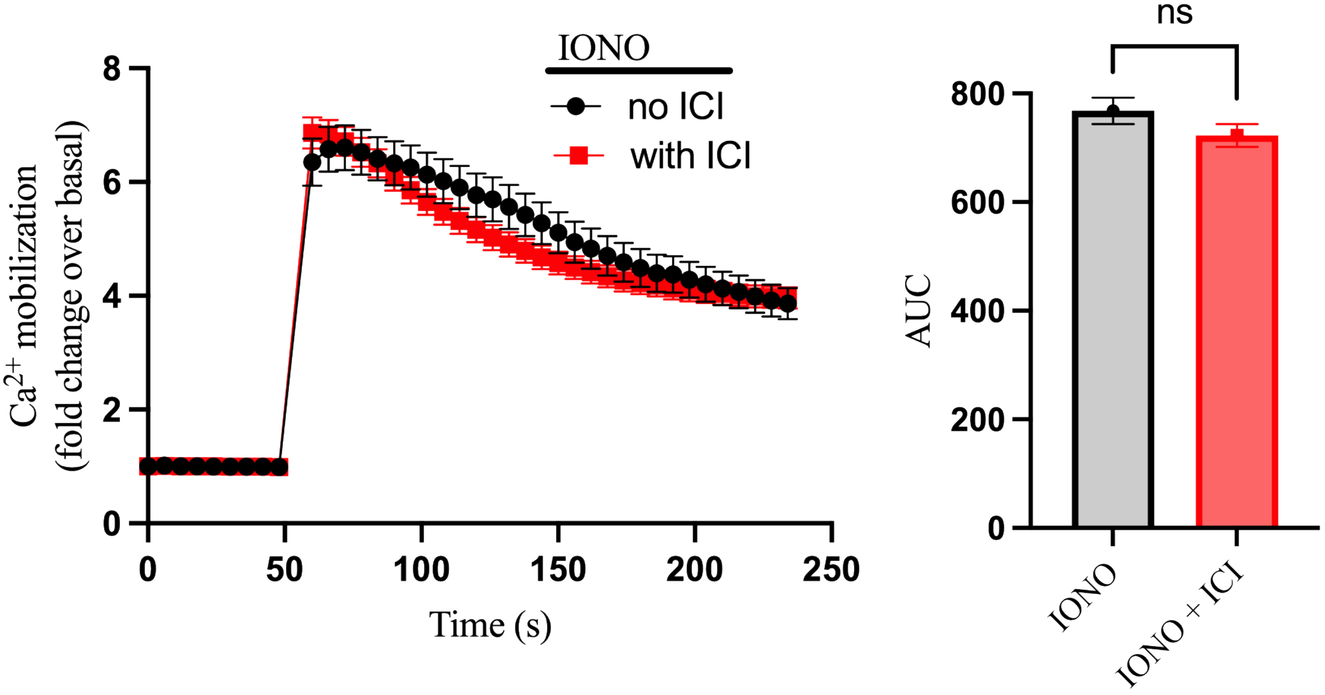

Figure S1. ***IONO-mediated Ca^{2+} mobilization with or without preincubation with ICI***

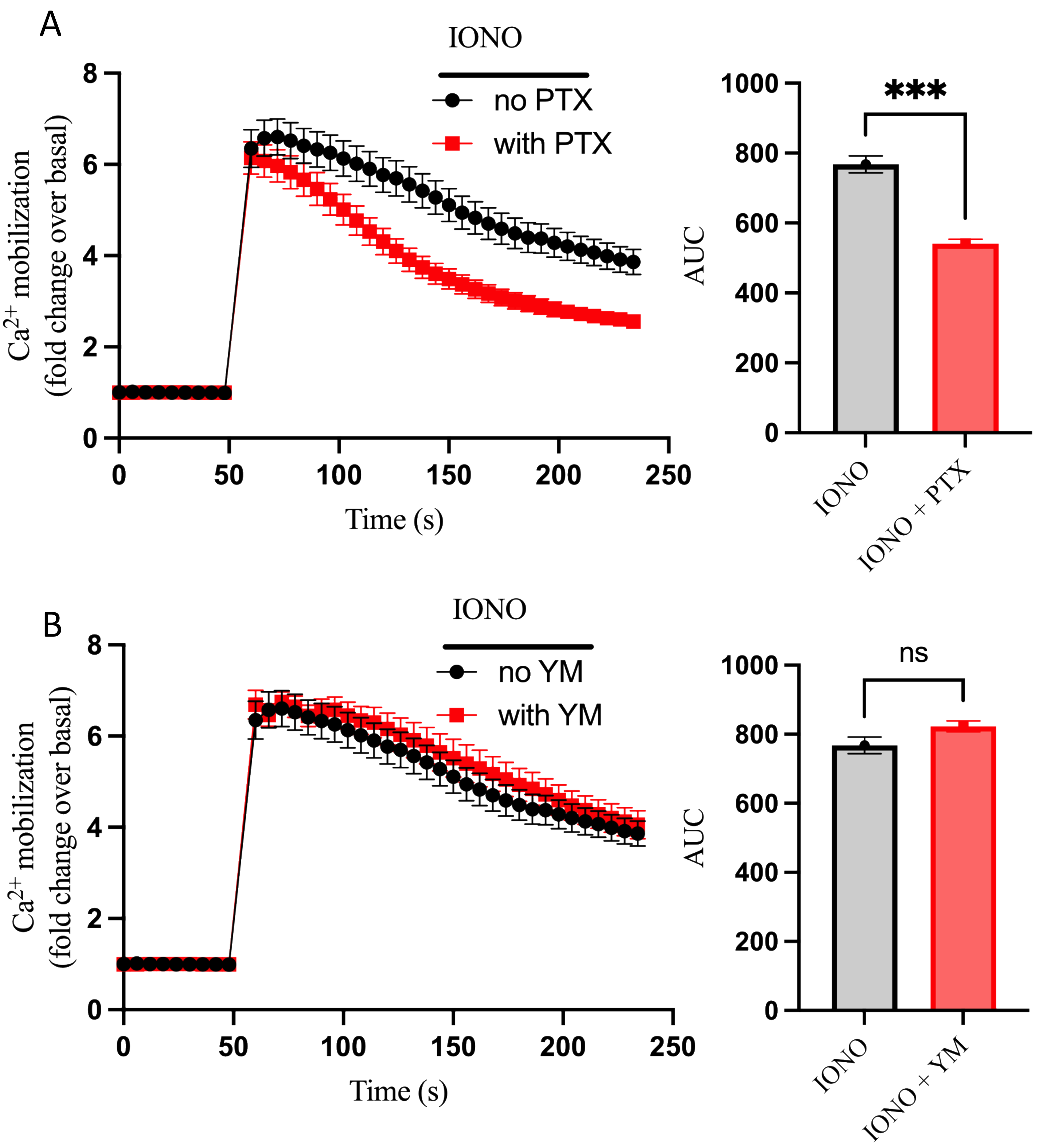

Figure S2. ***IONO-mediated Ca^{2+} mobilization with or without preincubation with PTX or YM***

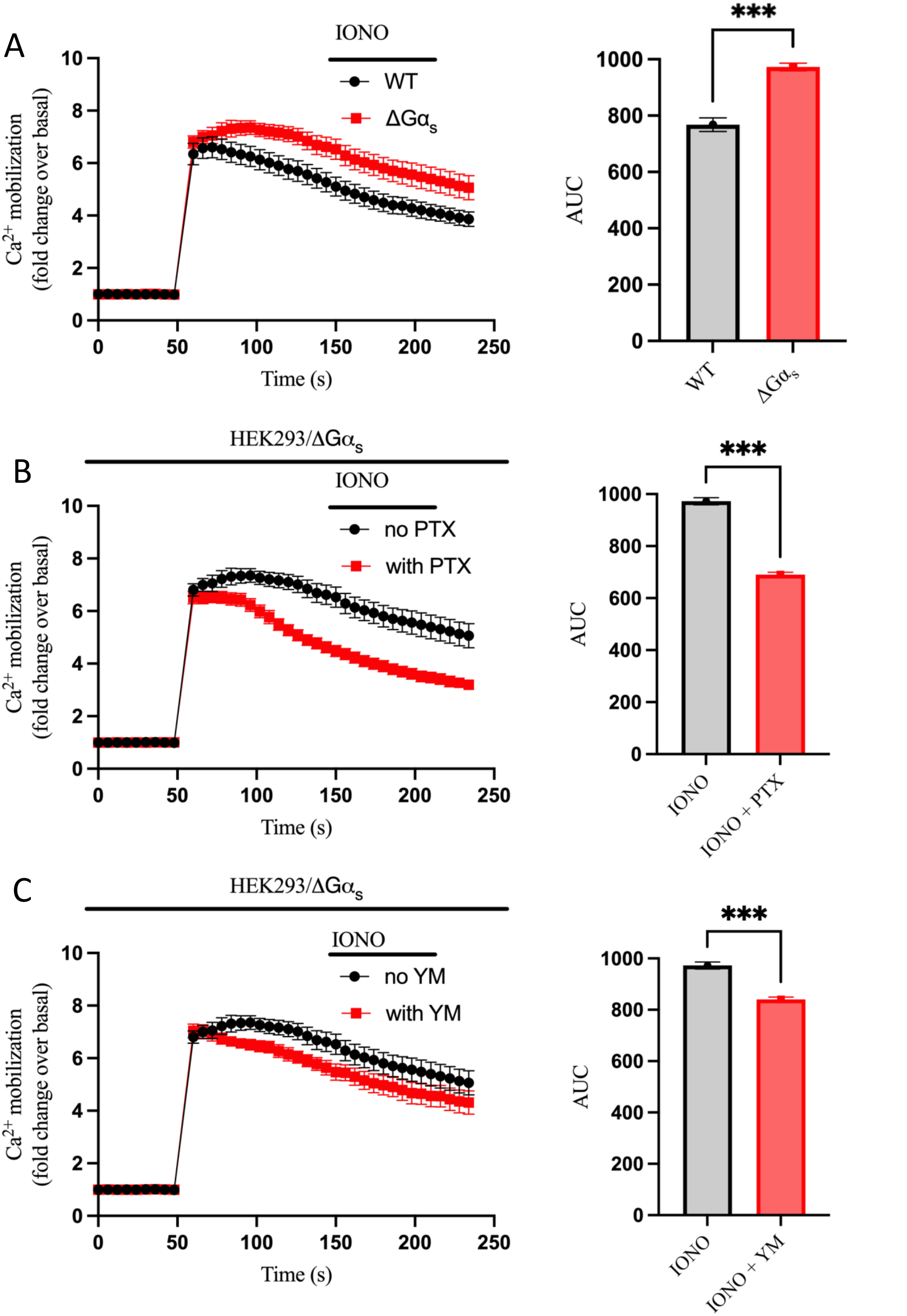

Figure S3. ***IONO-mediated Ca^{2+} mobilization in HEK293/ΔGα$_s$ cells***

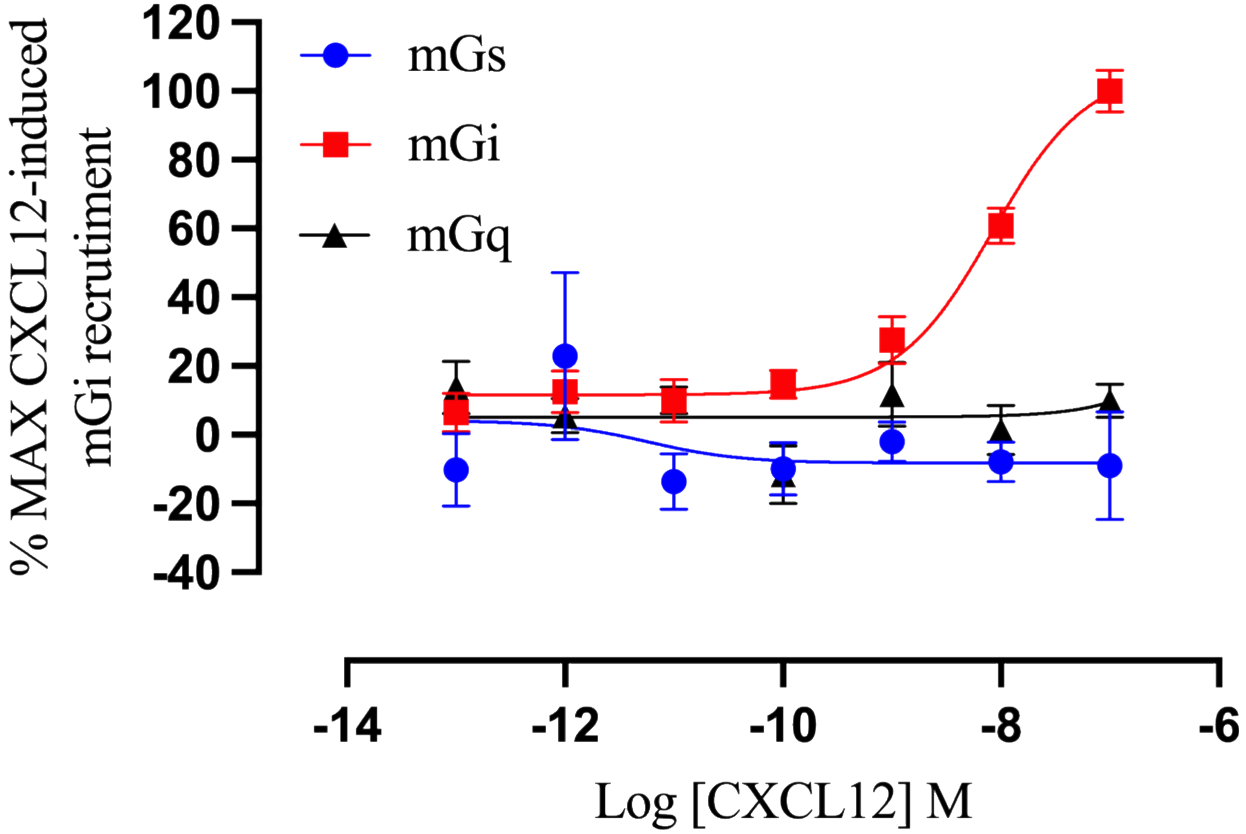

Figure S4. ***Specificity of the mini-G protein constructs***

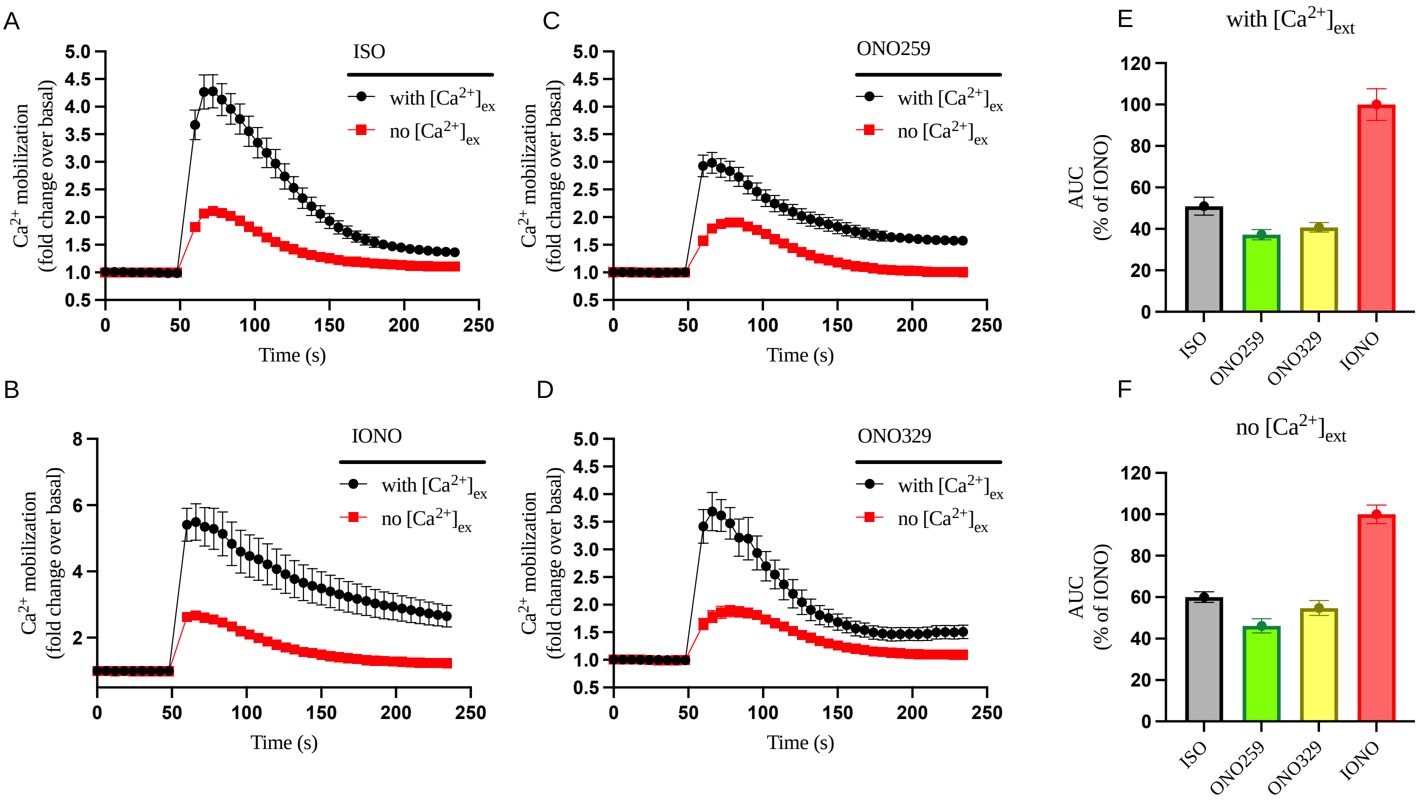

Figure S5. ***Agonist-promoted Ca^{2+} mobilization with or without extracellular Ca^{2+}***

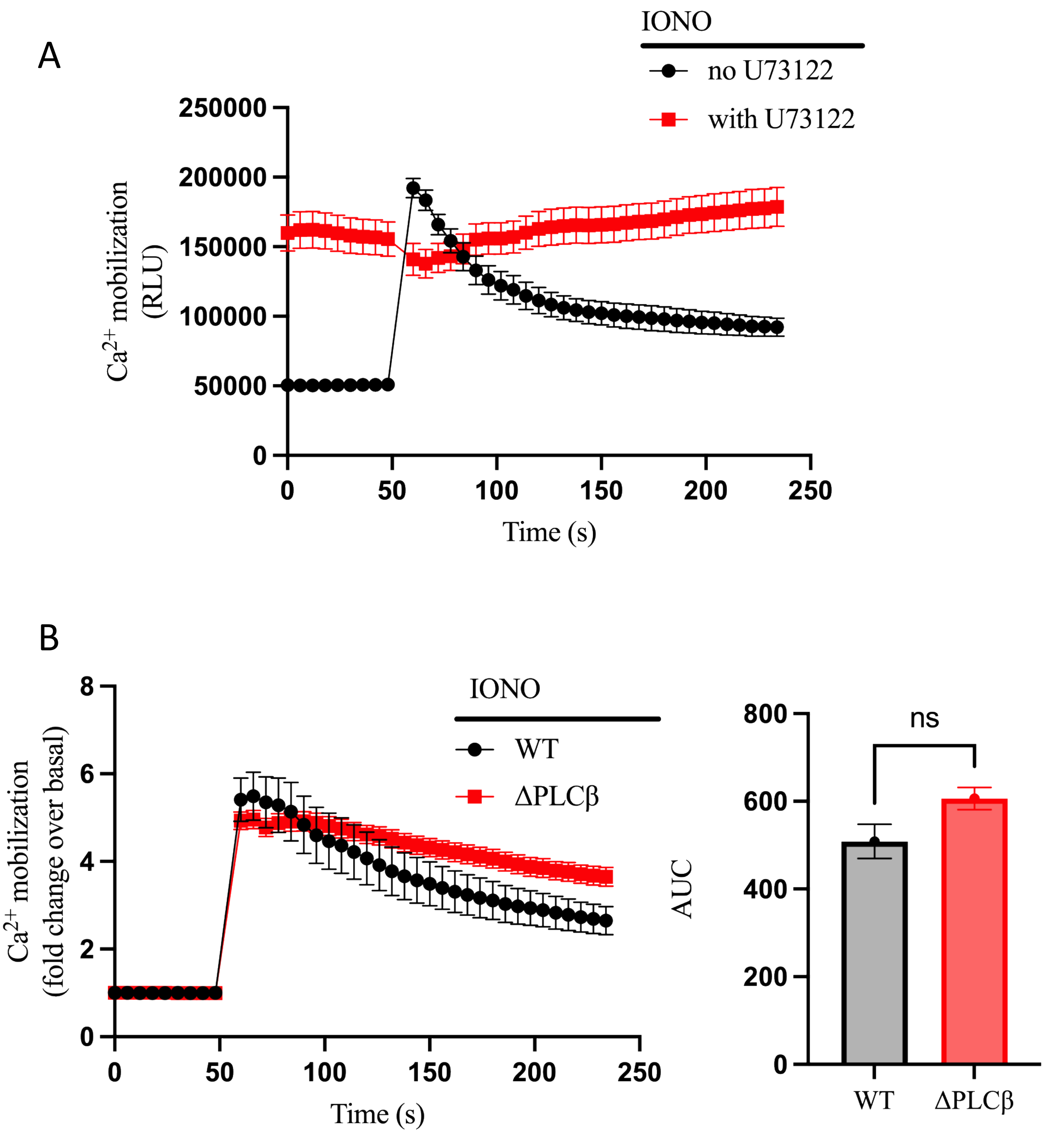

Figure S6: ***IONO-mediated Ca^{2+} mobilization with or without U73122 and in WT vs ΔPLCβ HEK293 cells.***
